# Normal Modes Expose Active Sites in Enzymes

**DOI:** 10.1371/journal.pcbi.1005293

**Published:** 2016-12-21

**Authors:** Yitav Glantz-Gashai, Tomer Meirson, Abraham O. Samson

**Affiliations:** Faculty of Medicine in the Galilee, Bar Ilan University, Safed, Israel; University of North Carolina at Chapel Hill, UNITED STATES

## Abstract

Accurate prediction of active sites is an important tool in bioinformatics. Here we present an improved structure based technique to expose active sites that is based on large changes of solvent accessibility accompanying normal mode dynamics. The technique which detects EXPOsure of active SITes through normal modEs is named EXPOSITE. The technique is trained using a small 133 enzyme dataset and tested using a large 845 enzyme dataset, both with known active site residues. EXPOSITE is also tested in a benchmark protein ligand dataset (PLD) comprising 48 proteins with and without bound ligands. EXPOSITE is shown to successfully locate the active site in most instances, and is found to be more accurate than other structure-based techniques. Interestingly, in several instances, the active site does not correspond to the largest pocket. EXPOSITE is advantageous due to its high precision and paves the way for structure based prediction of active site in enzymes.

## Author Summary

In this paper, we present an improved technique to predict active sites in enzymes. Our technique is based on changes of solvent accessibility that accompany normal mode dynamics. We assert the technique strength using several enzyme datasets with known catalytic residues. We show the technique successfully locates the active site in most cases, and consistently surpasses the accuracy of other techniques. We show how the technique is advantageous and paves the way for high precision prediction of active sites.

## Introduction

Prediction of functional sites in proteins is essential for a range of bioinformatics applications such as molecular docking, and structure based drug design. Traditional methods for predicting functional sites include three approaches: 1). The first approach uses sequence homology to find evolutionary conserved residues with functional activity. 2). The second approach utilizes structural homology with other proteins of known function to locate functional regions. 3). The third and last approach uses geometry and physico-chemical attributes of the protein structure and sequence to identify areas with functional activity.

Over the years, several techniques based on the third approach have been developed. These techniques include LIGSITE [1], POCKET [2], POCKET-FINDER [3], SURFNET [4], CAST [5], PASS [6], Cavity Search [7], VOIDOO [8], APROPOS [9], LigandFit [10], 3DLigandSite [11], MSPocket [12], Fpocket [13], McVol [14], Ghecom [15], PocketDepth [16], PocketPicker [17], VICE [18], as well as consensus techniques which use a combination thereof such as MetaPocket [19]. Other methods analyze the protein surface for pockets [20, 21], cavities [22–24], and channels [25] using pure geometric characteristics, and do not require any prior knowledge of the ligand or of sequence homology. Other computational techniques use geometric characteristics in combination with physico-chemical traits. Such methods include FOD [26], and Elcock [27] that analyze the hydrophobicity distribution under the assertion that functionally important residues are often in electrostatically unfavorable positions. Similarly, THEMATICS [28] uses geometric characteristics in combination with theoretical microscopic titration analysis, while the methods of Goodford [29] and Rupert et al. [30], and SiteHound [31] identify ligand binding sites based on analyses of the binding energies of probes placed on a grid around the protein. Another purely geometric method, EnSite, uses the proximity of catalytic residues to the molecular centroid to accurately detect the active sites of enzymes with high accuracy [32].

When used in combination with sequence and structure homology, geometric techniques are enhanced and prediction is improved. Some techniques use a vast combination of parameters ranging from conservation, residue type, accessibility, 2D structure propensity, cleft depth, B-factors, etc. to predict active site residues. Using such parameters, Gutteridge et al. predicted the location of active sites in enzymes using a neural network and spatial clustering [33]. Similarly Petrova et al. used Support Vector Machine with selected protein sequence and structural properties to predict catalytic residues [34]. In both cases, about 90% of the actual catalytic residues were correctly predicted. From these data it is clear, that one should rely on sequence and structure homology when possible, and over the past decade, multiple methods to detect binding sites and functional pockets based on geometric, structural, and genetic data were developed [35–39]. Several webservers of ligand binding sites have also been constructed and may be used to infer unknown ligand binding sites based on homology and other attributes such as Pocketome [40], FunFold [41], scPDB [42], IBIS [43], Multibind [44], fPop [45], and FINDSITE [46]. To date however, no comprehensive study comparing geometry based techniques has been performed.

Normal-mode analysis is one of the standard techniques for studying long time dynamics and, in particular, low-frequency motions. In contrast to molecular dynamics, normal-mode analysis provides a very detailed description of the dynamics around a local energy minimum. Even with its limitations, such as the neglect of the solvent effect, the use of harmonic approximation of the potential energy function, and the lack of information about energy barriers and crossing events, normal modes have provided much useful insight into protein dynamics. Over the past years, several techniques have been described to calculate large-scale motions using simplified normal-mode analysis [47–51]. Based on these techniques, several executable programs to calculate normal modes have been released, such as ElNemo [52], GROMACS [53], and STAND [49].

Recently, several studies have drawn attention to the allosteric effect of ligand binding on normal modes dynamics [54]. From these studies, a clear correlation between binding in the native site and perturbation of normal modes was identified. The same allosteric effect of ligand binding on molecular dynamics was also pointed out by Bhinge [55] and Ming [56] which proceeded to use molecular dynamics simulations in predicting ligand binding sites. It is based on these recent advances, that we became aware of the capacity of normal modes in predicting active sites.

**Fig 1:**
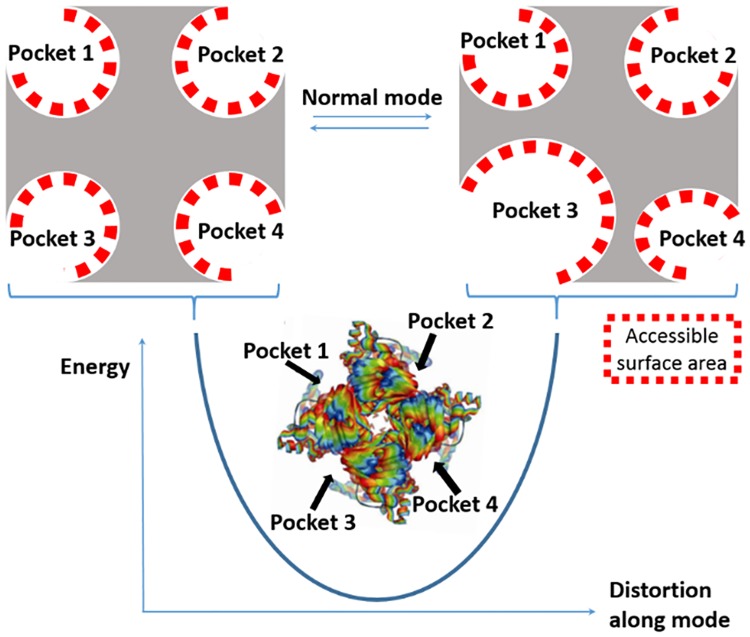
Accessible surface changes in normal modes. Upon distortion along a normal mode, different pockets experience different changes of accessible surface. In the shown example, the accessible surface of pockets 1 and 2 does not significantly change. However, the accessible surface of pocket 3 significantly changes to a larger extent than pocket 4. The solvent accessible surface is represented as red squares.

In this paper we present a novel structure based technique using normal modes to predict the location of active sites in enzymes. The technique exploits the normal mode opening and closing motion of enzymes and the accompanied change of solvent accessibility and highlights residues of the active site. The idea behind the presented technique is that active sites pockets become exposed in normal mode dynamics (Fig 1).

The hypothesis that active sites are surrounded by a shell of flexibility is not new and has been proposed in the dynamic lock-and-key model for biomolecular interactions. The shell of flexibility allows the enzyme to adapt to its ligand through an induced fit. The hypothesis was demonstrated in several studies notably by Weng et al. in a recent study on the flexibility of enzyme active sites [57], and less recently by Babor et al [58].

The technique which detects EXPOsure of active SITes through normal modEs is named EXPOSITE. The technique may also be used in association with other methods to rank geometrically calculated pockets according to their solvent exposure. First, the prediction strength of EXPOSITE is trained extensively in a dataset containing 133 enzymes with known active sites from the Catalytic Site Atlas (CSA) database [59]. Then, EXPOSITE is tested in a dataset containing 845 enzymes and found to be more robust than other structure-based techniques. EXPOSITE’s high success rate is valuable for structure-based identification of active sites and clearly shows the added value of using normal modes for finding active sites. The technique does not attempt to withdraw from the importance of using genetic data, and clearly, a combination of both structural and genetic data would be more useful for predicting active sites than any of them on their own.

## Methods

### Dataset assembly

To assemble a training dataset containing 133 enzymes with known active sites, enzymes were selected from the CSA database [59], version 2.2.1. The dataset enzymes were selected according to the following two criteria: 1). The enzyme active site is known from the literature (LIT), and not derived by homology. 2). The biologically active enzyme is composed of a single polypeptide chain and a single oligomerization state.

To assemble a test dataset containing 845 enzymes, enzymes were selected from the CSA database [59], version 2.2.1. The test dataset was compiled by extracting chain A of all LIT entries that were not included in the 133 training dataset. These two datasets were used for training and testing EXPOSITEs prediction consistency respectively.

### Normal mode calculations

To calculate normal modes of the dataset enzymes, two programs were utilized namely STAND [49] and ElNemo [52] and were run locally. For STAND, both real normal modes (REA) and Tirion modes (TIR) were calculated. For speed, the STAND option of coarse graining, 1 point (1 pt), which accelerates the calculations yet does not flaw the results was used, and defaults values of deformation amplitude were used. For ElNemo, default values of DQMIN -100 and DQMAX 100 were utilized. The DQMIN and DQMAX parameters correspond to the deformation amplitude in the direction of a single normal mode. For both STAND and ElNemo, deformation amplitudes were not scaled, and the same amplitude produces smaller deformation for larger molecules. For both STAND and ElNemo, only the 10 non-trivial lowest frequency modes were calculated. For each of these 10 modes, 40 PDB files were generated by STAND and 10 PDB files were generated by ElNemo all distorted along the particular mode. The two methods are very different in that STAND (REA) minimizes the structure and then calculates modes in φ and ψ torsion angle space whereas STAND (TIR) and ElNemo avoid minimization by using Tirion modes [50] and then calculate modes in Cartesian coordinate space. For STAND, the opposite extremes of the harmonic motion were empirically chosen as the 1^st^ and 14^th^ structure out of 40 respectively. At these extremes, the structures look fully “distorted” from each other. For ElNemo, the opposite extremes of the harmonic motion are the 1^st^ and 10^th^ structure out of 10.

### Solvent accessible surface calculations

To calculate the solvent accessible surface (SAS) area of amino acids in the generated PDB files, the DSSP program was used [60]. For each mode, SAS for each residue in the two structures at opposite extremes of the harmonic motion were calculated, and the absolute change of SAS between the extreme mode distortions, |ΔSAS| was used.

### Pocket calculation

To calculate pockets, LIGSITE [61] was run locally using default parameters. In each case, the 10 largest pockets were calculated and the pocket center as well as the pocket size were collected.

### Prediction of active site

The predicted active site was defined as the geometrical center (centroid) of the Cα coordinates of all residues with a solvent exposure |ΔSAS|, in the range 20-40Å^2^

The observed active site was defined as the geometrical center (centroid) of the Cα coordinates of the active site residues specified in the CSA database [59].

The predicted and observed active sites were represented each by a single coordinate in Cartesian space. The distance between these two coordinates was defined as the distance between the predicted and observed sites.

The success of a prediction was based on the distance between the predicted and observed sites in the training and test datasets. If the distance between the predicted and observed sites was less than 12Å, then a prediction was considered successful. Conversely, if the distance was larger than 12Å, then a prediction was deemed incorrect.

In the special case of the PLD dataset and for easy comparison with other techniques, a prediction was considered successful if any atom coordinate of the ligand was within 4Å of the predicted site. If no atom coordinate of the ligand was within 4Å of the predicted site, then the prediction was considered wrong.

### Comparison with other techniques

To compare EXPOSITE with other techniques, several software were run on all datasets namely, the training dataset of 133 enzymes, and the testing dataset of 845 enzymes, as well as a dataset containing 48 proteins derived from the PLD dataset [62] and engineered by Huang et al [61]. First, each of the following software was downloaded: LIGSITE, CAST, PASS, and SURFNET. For EnSite, no software was available, and the script was reconstructed based on the algorithm described in the original paper [32]. Then, each of the software was run locally on a PC running under Windows or Linux. In the case of the training and test datasets (which lacks ligands), a prediction was considered successful if the predicted and observed active site were less than 12Å apart. In the case of the PLD dataset (which contains ligands), a prediction was considered successful if the predicted active site was less than 4Å apart from any ligand atom.

## Results

### Dataset assembly

To reliably assess the success rate of our technique in an sizeable ensemble, two datasets were assembled from the CSA database [59]. The CSA database contains 23,265 enzymes with known active sites. Of these, only 845 had an active site known from the literature (LIT), and comprised the test dataset. Of these, only 133 were composed of a single chain that is biologically active as a monomer in a single oligomerization state, and comprised the training dataset. The PDB IDs of the 133 selected enzymes of the training dataset are listed in S1 Table. The PDB IDs of the 845 enzymes of the test dataset are listed in S2 Table. To test for homology within the datasets, the enzyme commission (EC) numbers were retrieved. Although, some homologues were found within a single dataset, no homologues were found between the training and test dataset.

### Calculation of pockets and solvent accessible surface

A number of programs were tested to calculate geometric pockets of biomolecular structures, i.e. POCKET [2], LIGSITE [1], POCKET-FINDER [3], SURFNET [4], CAST [5], PASS [6]. The program LIGSITE^CSC^ [61] provides a list of pocket centers and size in a PDB format and was subsequently utilized in all our calculations.

Surprisingly, there are significant differences between SAS of residues calculated by DSSP and other techniques such as ENCAD, CNS, and Accelrys. These differences arise from the different approaches used in calculating SAS. Nonetheless, when calculating the change of surface areas, ΔSAS, these differences cancel out and all programs produce comparable ΔSAS values.

### EXPOSITE training

Biologically relevant modes are not always represented in the lowest frequency modes. Sampling more data, i.e. by calculating more modes could provide better results. Similarly, changing the |ΔSAS| thresholds could also lead to a higher success rate by allowing more exposure data to be included. To test this assertion and optimize the success rate of EXPOSITE the following parameters were varied: the threshold value of |ΔSAS| and the number of normal modes sampled. The number of modes sampled was varied from 0 to 10 and the |ΔSAS| minimum and maximum thresholds were changed from 0 to 60 Å^2^.

As seen in S4 Table, the optimal |ΔSAS| thresholds for ElNemo were around 20 and 40 Å^2^ respectively. Below the threshold of 10 Å^2^, normal exposure fluctuations contribute little to EXPOSITE’s accuracy. Above the threshold of 40 Å^2^, exposure changes arise from the normal mode tip effect (bond breaking and exaggerated exposure) and contribute little to the EXPOSITE accuracy. For STAND, the optimal |ΔSAS| threshold values were 20 and 40 Å^2^ respectively. This difference of |ΔSAS| thresholds between STAND and ElNemo is due to the fact that STAND uses coarse graining, inherently reducing the surface area, whereas ElNemo does not. STAND uses coarse graining and represents each amino acid with a single bead, while ElNemo uses a heavy atoms representation. In both cases, the maximum deformation amplitude were not chosen and default values were used. Also, the maximum deformation amplitude was not scaled in this study.

The optimal number of mode sampling peaks to a plateau around modes 8, 9, and 10 for both STAND and ElNemo (S5 Table). Below this sampling number important information is lost. Intriguingly, when using no threshold for |ΔSAS|, the accuracy of EXPOSITE is consistently 86%, no matter how many modes are sampled.

### Correlation of predicted and observed active site in 133 enzyme training dataset

**Fig 2:**
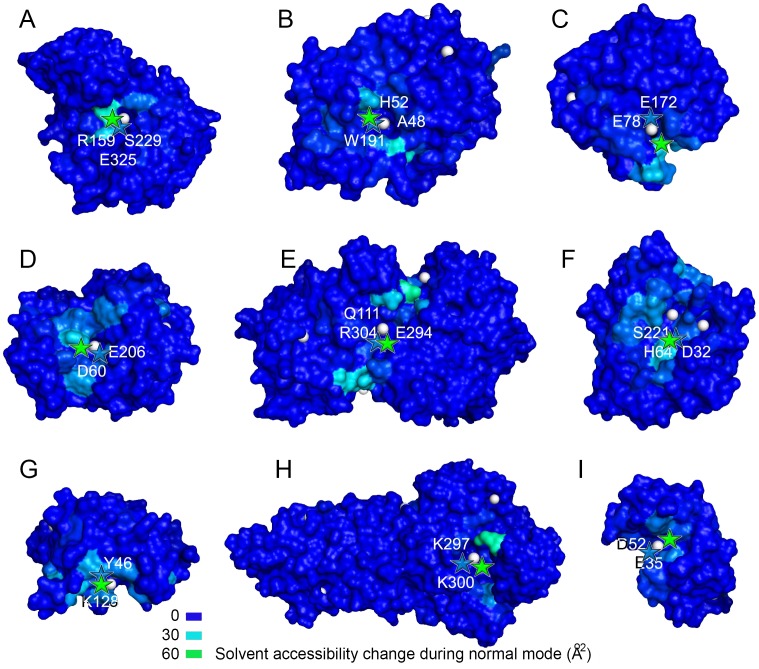
Solvent accessibility changes in normal modes highlight active sites of enzymes. Shown are nine EXPOSITE predictions for the enzymes (A) 1mbb, (B) 1dj1, (C) 1bvv, (D) 1pgs, (E) 1pmi, (F) 1sca, (G) 1lba, (H) 1a8h, and (I) 132l of the training dataset. The predicted and observed active sites are indicated by green and blue stars, and LIGSITE pockets are displayed as white spheres. In cyan and green are residues experiencing large accessibility changes in normal modes, and in blue, are residues experiencing little or no exposure change. Note that the predicted and observed active site are separated by less than 12Å. The figure was prepared using Pymol.

EXPOSITE uses solvent accessibility changes in normal-modes to predict the location of active sites in enzymes. As seen in Fig 2, residues experiencing large accessibility changes (colored cyan and green) are likely to be found in proximity to active site residue (shown in text). In contrast, residues experiencing little exposure change (colored blue) are less likely to be found in vicinity of the active site. The proximity between residues experiencing large |ΔSAS| and the experimentally observed active site residues is an indicator of the precision of EXPOSITEs prediction.

On average, the predicted and observed active sites in the training dataset are separated by 7.9 Å, and a standard deviation of 4.4 Å (S1 Fig).

The maximum success rate of EXPOSITE in the training dataset consisting of 133 enzymes was 92%. Curiously, in the training dataset, the binding pocket coincides mostly with the largest pocket (82%) but not always (18%). This finding accounts for the pitfall of other techniques which rely on pocket size only for ranking.

Also interesting is the fact that no active site was found in pockets with a size less than 7 Å^3^. Such pockets are too small to accommodate ligands and validate our convention of discarding them as insignificant.

### Correlation of predicted and observed active site in test dataset

**Fig 3:**
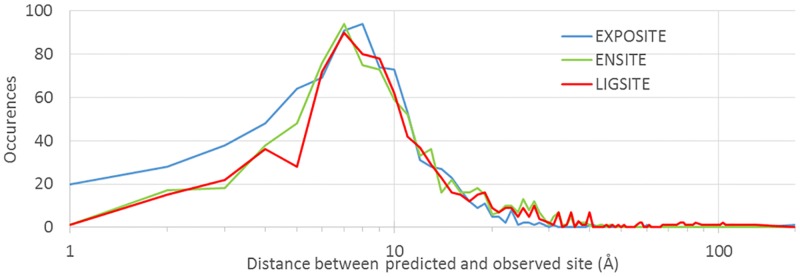
Line graph of distances between the predicted and observed active sites in the 845 enzyme test dataset. The distances between the predicted and observed sites are plotted in blue, in green, and in red for EXPOSITE, ENSITE, and LIGSITE respectively. The distribution of distances is shown on a logarithmic scale, and emphasizes the added value of normal modes for prediction of active sites.

Shown in Fig 3 is a histogram of distances between the predicted and observed active sites in the 845 enzyme test dataset. In this dataset, the predicted and observed catalytic sites are separated by an average of 9.2 Å, 11.5 Å, and 14.1 Å for EXPOSITE, ENSITE, and LIGSITE respectively (Fig 3). Significantly, if a successful prediction is arbitrarily defined by a distance cutoff of 4 Å, then the number of hits of EXPOSITE (16.1%) is almost double that of ENSITE (8.7%). Similarly, if a successful prediction is arbitrarily defined by a distance cutoff of 3 Å, then the number of hits of EXPOSITE (10.4%) is 2.4 times that of ENSITE (4.3%).

To test the robustness of EXPOSITE, we tested its success rate in a dataset containing 845 enzymes (S2 Table). Not surprisingly, the success rate is much lower than in the 133 enzyme dataset. Reliably however, EXPOSITE is better that EnSite in predicting the active site by >2%. The sharp decrease of prediction success rate in the 845 enzymes dataset is not surprising, as the dataset does not discriminate between real homomonomeric enzymes with high success rates, and homomultimeric enzyme assemblies with low success rates (close to 0). Even if statistically robust, the large 845 enzyme dataset does not reflect the real success-rate of prediction techniques, and the smaller 133 enzyme dataset should be regarded as a more representative alternative. The large 845 enzymes dataset is too diverse, and demonstrates the difficulty in assembling representative datasets.

### Correlation of predicted and observed active site in PLD dataset

**Fig 4:**
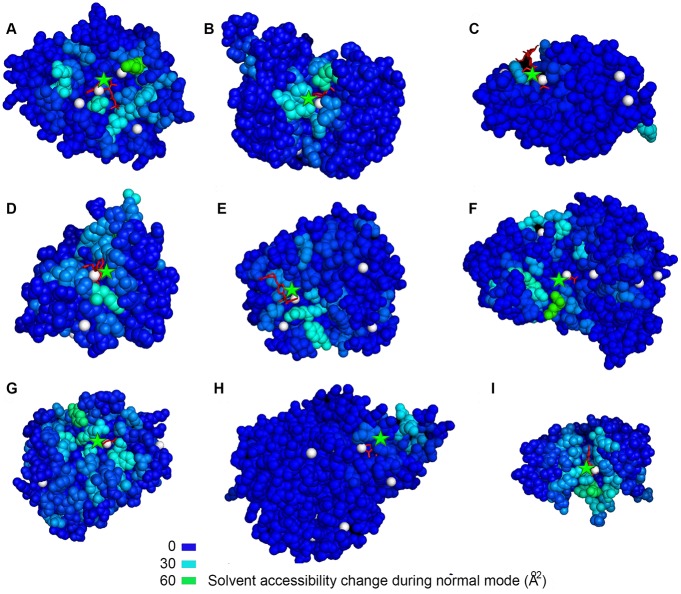
Solvent accessibility changes in normal modes highlight the ligand binding site of proteins. Displayed are EXPOSITE predictions of nine proteins (A) 1inc, (B) 1bid, (C) 1hew, (D) 1hfc, (E) 1imb, (F) 1mrg, (G) 1mtw, (H) 1ulb, and (I) 1rob from the PLD database. The predicted and observed binding sites are indicated by green stars and red ligands respectively, and LIGSITE pockets are displayed as white spheres. In cyan and green, are residues displaying large changes of accessibility in normal modes, and in blue, are residues which display little or no change of exposure. Note that the ligand (in red) is within 4Å of the predicted site (green star). The figure was prepared using Pymol.

EXPOSITE highlights the binding site of proteins of the Protein Ligand Dataset (PLD) published elsewhere [62]. Shown in Fig 4 (and in S2 Fig) are a few examples of ligand binding site prediction. Residues experiencing large accessibility changes (colored green) are likely to be found in proximity to the ligand (colored red), whereas residues experiencing little exposure change (colored blue) are further away. The proximity of residues with large accessibility changes and residues of the observed active site is a success indicator of EXPOSITEs predictions.

On average, the predicted and observed centers in the protein PLD dataset are separated by 7 Å with a standard deviation of 3.3 Å. Intriguingly, the separation in the PLD dataset is smaller than that of the CSA dataset by almost 1 Å, and it is probably a flaw due to the handpicked nature of the PLD dataset.

### Comparison to other techniques

To accurately and robustly compare EXPOSITE with other techniques, all other software were run on all datasets namely the training dataset of 133 enzymes, the testing dataset of 845 enzymes. A prediction was considered accurate if the distance between the predicted and observed sites was less than 12Å. If the distance was larger than 12Å, then a prediction was considered inaccurate. The calculated prediction accuracies are listed in Table 1.

**Table 1:** Percent success rate of predictions.

Method	Training dataset (133 enzymes)	Test dataset (845 enzymes)
**EXPOSITE**	92	74
**EnSite**	86	72
**LIGSITE**^***csc***^	69	59
**CAST**	55	50
**PASS**	60	45
**SURFNET**	49	42

When compared to other geometric techniques EXPOSITE is advantageous due to its high success rate. As seen in Table 1, EXPOSITE is only slightly better than EnSite at predicting active sites and EnSite is still superior to EXPOSITE in speed as it is ingenious in simplicity. Also note that prediction of binding sites in unbound proteins is less successful than that of ligand-bound proteins simply because the ligands occupy and expose the binding site through induced fit thereby easing its identification.

**Fig 5:**
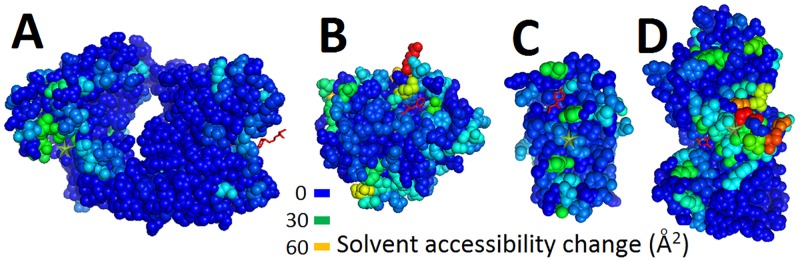
Failures to highlight the binding site of proteins. Displayed are EXPOSITE predictions of four proteins (A) 1igj, (B) 3gch, (C) 3mth, (D) and 2tmn, from the PLD database. The predicted and observed binding sites are indicated by green stars and red ligands respectively. In orange, cyan, and green, are residues displaying large changes of accessibility in normal modes, and in blue, are residues which display little or no change of exposure. Note that EXPOSITE failed to predict the binding site in these cases due to multiple backbone breaks resulting in unusual modes (i.e. 3mth, 3gch), and to odd shaped protein structure (i.e. 1igj). The figure was prepared using Pymol.

To accurately and robustly compare EXPOSITE with other techniques, all other software were run on the bound and unbound PLD dataset [61]. A prediction was considered accurate if any ligand atom was within 4Å of the predicted site. If no ligand atom was within 4Å of the predicted site, then the prediction was considered inaccurate. The calculated prediction accuracies are listed in Table 2. The data for EXPOSITE and Ensite is reported by us, the data for VICE was reported by Tripathi et al [18], the data for Fpocket was reported by Le Guilloux et al. [13], the data for PocketPicker was reported by Weisel et al. [17], the data for LIGSITEcs, CAST, PASS and SURFNET were first reported by Huang et al. [63]. Please note that EXPOSITE is not always successful, such as in the case of PDB 1igj, 3gch, 3mth, and 2tmn as may be seen in Fig 5.

**Table 2:** Comparison of success rate for 48 complexed and 48 unbound protein structures.

Method	Protein Ligand dataset (48 enzymes)	
	Unbound	Bound
EXPOSITE	86	92
Ensite	84	86
VICE	83	85
Fpocket	69	83
PocketPicker	69	72
LIGSITE^cs^	60	69
CAST	58	67
PASS	60	63
SURFNET	52	54

Intriguingly, the classically accepted metric for binding site prediction is 4Å, and we used this metric in the classical PLD dataset when comparing the classical performance of EXPOSITE, Ensite, VICE, Fpocket, PocketPicker, LIGSITEcs, CAST, PASS and SURFNET (Table 2). However, in the unclassical training and test datasets which were never tested before, we relied on an unclassical distance of 12Å. The training and test datasets contain 20 times more proteins than the hand-picked PLD dataset, and if the classical distance of 4Å was used, then the performance of all techniques sank drastically. To maintain good performances for all techniques in the training and test datasets, the classically accepted metric for binding site prediction was raised to an unclassical 12Å.

Generally speaking, the success rate in the handpicked PLD dataset is higher than in the non-handpicked 845 test dataset. This discrepancy suggests that the PLD dataset was not randomly picked, and could artificially increase prediction success rates.

### EXPOSITE ranks active site pockets

EXPOSITE’s feature, of highlighting active sites is very useful for ranking pockets. Indeed, the technique is capable of ranking enzyme pockets according to their degree of exposure in normal mode dynamics. This ranking enables EXPOSITE to choose the correct binding pockets from a list of pockets calculated by LIGSITE. The assumption that the active site pockets is usually in the largest pocket [1, 4, 64] is being used by several pocket detection programs and the top site is generally the largest one. However, this assumption is not always true and in several instances, the active site corresponds to the second, third, or fourth largest pocket.

## Discussion

### EXPOSITE rationale

The rationale behind the success rate of EXPOSITE is fairly simple. For proper enzyme activity, protection from the surrounding water is often necessary as shown by normal modes closure of the active site. Proteins in general and enzymes in particular often act as environment protectors. They envelop substrates to catalyze chemical reaction that would otherwise not take place in aqueous solution. They conceal prosthetic groups to coordinate binding thus increasing affinity which is negligible in water. They act as small shielding cases displaying alternating motions of opening and closing to allow ligand entrance and protection respectively. Throughout this motion, protein residues located at various distances from the active site are exposed to the solvent to a different degree. Residues in proximity to the active site are exposed more than those faraway. This idea lays down the foundation for EXPOSITE suggesting the pocket closest to the maximum exposure center is the active site.

The change in solvent accessibility between the X-ray structure and the largest deformation of either of the normal mode extremes could also have been used. However, the maximum effect of motion is observed between the two extremes which vibrate around the X-ray structure corresponding to a local minimum.

### EXPOSITE parameters

EXPOSITE takes into account several parameters such as accessibility change in normal modes, centroid distance from pockets, as well as pocket size. Normal modes by their own virtue take into account more parameters such as contact network and distances. Together, these parameters resemble those used in neural network techniques [33, 34] where they are analogous to accessibility, cleft depth, B-factors, etc… As much as these techniques seem different, the analogy between the parameters is astounding.

### Coarse graining does not decreases EXPOSITE success rate

The success rate is not affected by the different types of normal modes techniques, STAND and ElNemo. The success rate remains unchanged even when STAND and ElNemo are used in different combinations with accessibility calculators (i.e. ElNemo with ENCAD accessibility calculator [65]. The success rate does not originate from the difference in the atomic representation used by ElNemo and STAND. In fact, when running STAND in full-atom representation the success rate remains unchanged. These data indicate that coarse graining which ignores the amino acid type and accessible surface does not influence the success rate of EXPOSITE. In fact, adding heavy atoms to the PDB files generated by STAND also does not decrease the success rate of EXPOSITE. We conclude that coarse-graining and accessibility calculation methods do not affect the success rate of EXPOSITE.

### Caveats of EXPOSITE

Care should be taken when using our technique on structures composed of several domains. Practical interpretation of normal modes of multi-domain structures tend to be problematic in the sense that bending and twisting of one domain relative to another tend to overshadow modes with biological meaning. One way to circumvent this problem is to run normal modes of single domains to predict its active site. We excluded multi chain enzymes which are biologically active in oligomeric states from our CSA dataset. Similarly, care should be taken when using EXPSOITE on structures with elongated termini or exceedingly flexible loops. Such structures often present odd normal modes around these areas which tend to overshadow modes with biological meaning. Some strongly recommended ways to circumvent the problem of exaggerated motion is simply to clip out (or edit out) the stretches and rerun normal mode computation or to set an upper value for the cutoff of |ΔSAS| of 75 Å^2^ when calculating modes with ElNemo (40 Å^2^ for STAND). The cutoff should minimize the effect of loose and flexible termini with exaggerated exposure change. A complete list of success and failures is provided in S6 and S7 Tables.

### Binding site vs. active site

A distinction should be made between the concepts “binding site” and “active site”. Usually, an active site is found in a single copy in an enzyme, while binding sites may be present in multiple copies in proteins. Thus, prediction of active sites and ligand binding sites are very different, and whereas only one prediction is correct for enzymes, several predictions are correct for proteins. To complicate things further, some enzymes are composed of multiple chains, each equipped with a distinct active site, and so much care should be taken so as not to over interpret a prediction. As a rule of thumb structure based predictions (EXPOSITE, EnSite, etc) are more accurate in single chain polypeptide enzymes.

### Absence of correlation between pocket size, substrate size, number of residues with high accessibility change, and number of active site residues

In an attempt to correlate between pocket size and active site, the following parameters of active site were calculated in the PLD dataset: 1). The number of Cα atoms of the active site was derived from the CSA database. 2). The number of heavy atoms in the substrate was calculated from the PLD database. 3). The number of residues of with high accessibility change was calculated from EXPOSITE. 4). The size of the predicted pocket in Å^3^ was from LIGSITE. These parameters all reflect on the size of the active site yet there is no obvious correlation among them. There was no correlation (R = 0.12) between pocket size and the number of active site residues. This is partially due to fractionation of active sites into adjacent pocket (POK) which decrease “real” active site size. This fractioning of active sites is a problem often encountered in pocket calculating programs. Adjoining sizes of vicinal pockets did not improve the correlation significantly.

## Conclusion

Over the past years normal modes have enjoyed a revival. In this article, the biological relevance of normal modes is illustrated in a new technique. The presented technique exposes active sites of enzymes with high success rates. As pocket detection methodologies normal mode techniques improve so will our technique. In the future, EXPSOITE is expected to become publicly available as a basic tool (website and/or program) for predicting active sites of enzymes. The Perl code used in this study is freely available in the supplementary data. Note that DSSP, LIGSITE, ElNemo, and/or STAND must be obtained from third parties, and that the time bottleneck of the method is normal mode calculation.

## Supporting Information

S1 Fig Histogram of distances between predicted and observed active sites in the 133 enzyme training dataset. The distribution of distances between the predicted and observed active sites is shown. Note that 92% of the predictions fall within 12 Å of the observed active site.
(TIF)

S2 Fig Solvent accessibility changes in normal modes highlight active sites of enzymes. Shown are four additional EXPOSITE predictions for the enzymes (A) 2pk4, (B) 1ulb, (C) 1stp, and (D) 1apu of the PLD dataset. The predicted and observed binding sites are indicated by green stars and red ligands respectively, and LIGSITE pockets are displayed as white spheres. In cyan and green, are residues displaying large changes of accessibility in normal modes, and in blue, are residues which display little or no change of exposure. Note that the ligand (in red) is within 4Å of the predicted site (green star). The figure was prepared using Pymol.
(TIF)

S1 Table List of 133 enzyme training dataset. (DOCX)

S2 Table List of 845 enzyme testing dataset. (DOCX)

S3 Table List of 48 proteins dataset derived from the Protein Ligand Database (PLD) by Huang et al. (DOCX)

S4 Table Training of EXPOSITE using different solvent accessible cutoffs in the 133 enzyme test dataset. (DOCX)

S5 Table Training of EXPOSITE using different numbers of modes in the 133 enzyme test dataset. (DOCX)

S6 Table List of success and failures of EXPOSITE in the 133 enzyme dataset. (DOCX)

S7 Table List of success and failures of EXPOSITE in the 845 enzyme dataset. (DOCX)

S1 Code Perl code used in the study. (ZIP)

## References

[1] Hendlich M., Rippmann F., and Barnickel G., LIGSITE: automatic and efficient detection of potential small molecule-binding sites in proteins. J Mol Graph Model, 1997 15(6): p. 359–63, 389 9704298 10.1016/s1093-3263(98)00002-3

[2] Levitt D.G. and Banaszak L.J., POCKET: a computer graphics method for identifying and displaying protein cavities and their surrounding amino acids. J Mol Graph, 1992 10(4): p. 229–34. 1476996 10.1016/0263-7855(92)80074-n

[3] Laurie A.T. and Jackson R.M., Q-SiteFinder: an energy-based method for the prediction of protein-ligand binding sites. Bioinformatics, 2005 21(9): p. 1908–16. 10.1093/bioinformatics/bti315 15701681

[4] Laskowski R.A., SURFNET: a program for visualizing molecular surfaces, cavities, and intermolecular interactions. J Mol Graph, 1995 13(5): p. 323–30, 307–8. 8603061 10.1016/0263-7855(95)00073-9

[5] Liang J., Edelsbrunner H., and Woodward C., Anatomy of protein pockets and cavities: measurement of binding site geometry and implications for ligand design. Protein Sci, 1998 7(9): p. 1884–97. 10.1002/pro.5560070905 9761470 PMC2144175

[6] Brady G.P. Jr. and Stouten P.F., Fast prediction and visualization of protein binding pockets with PASS. J Comput Aided Mol Des, 2000 14(4): p. 383–401. 10815774 10.1023/a:1008124202956

[7] Ho C.M. and Marshall G.R., Cavity search: an algorithm for the isolation and display of cavity-like binding regions. J Comput Aided Mol Des, 1990 4(4): p. 337–54. 2092080 10.1007/BF00117400

[8] Kleywegt G.J. and Jones T.A., Detection, delineation, measurement and display of cavities in macromolecular structures. Acta Crystallogr D Biol Crystallogr, 1994 50(Pt 2): p. 178–85. 10.1107/S0907444993011333 15299456

[9] Peters K.P., Fauck J., and Frommel C., The automatic search for ligand binding sites in proteins of known three-dimensional structure using only geometric criteria. J Mol Biol, 1996 256(1): p. 201–13. 10.1006/jmbi.1996.0077 8609611

[10] Venkatachalam C.M., et al, LigandFit: a novel method for the shape-directed rapid docking of ligands to protein active sites. J Mol Graph Model, 2003 21(4): p. 289–307. 12479928 10.1016/s1093-3263(02)00164-x

[11] Wass M.N., Kelley L.A., and Sternberg M.J., 3DLigandSite: predicting ligand-binding sites using similar structures. Nucleic Acids Res, 2010 38(Web Server issue): p. W469–73. 10.1093/nar/gkq406 20513649 PMC2896164

[12] Zhu H. and Pisabarro M.T., MSPocket: an orientation-independent algorithm for the detection of ligand binding pockets. Bioinformatics, 2011 27(3): p. 351–8. 10.1093/bioinformatics/btq672 21134896

[13] Le Guilloux V., Schmidtke P., and Tuffery P., Fpocket: an open source platform for ligand pocket detection. BMC Bioinformatics, 2009 10: p. 168 10.1186/1471-2105-10-168 19486540 PMC2700099

[14] Till M.S. and Ullmann G.M., McVol—a program for calculating protein volumes and identifying cavities by a Monte Carlo algorithm. J Mol Model, 2010 16(3): p. 419–29. 10.1007/s00894-009-0541-y 19626353

[15] Kawabata T., Detection of multiscale pockets on protein surfaces using mathematical morphology. Proteins, 2010 78(5): p. 1195–211. 10.1002/prot.22639 19938154

[16] Kalidas Y. and Chandra N., PocketDepth: a new depth based algorithm for identification of ligand binding sites in proteins. J Struct Biol, 2008 161(1): p. 31–42. 10.1016/j.jsb.2007.09.005 17949996

[17] Weisel M., Proschak E., and Schneider G., PocketPicker: analysis of ligand binding-sites with shape descriptors. Chem Cent J, 2007 1: p. 7 10.1186/1752-153X-1-7 17880740 PMC1994066

[18] Tripathi A. and Kellogg G.E., A novel and efficient tool for locating and characterizing protein cavities and binding sites. Proteins, 2010 78(4): p. 825–42. 10.1002/prot.22608 19847777 PMC2811767

[19] Zhang Z., et al, Identification of cavities on protein surface using multiple computational approaches for drug binding site prediction. Bioinformatics, 2011 27(15): p. 2083–8. 10.1093/bioinformatics/btr331 21636590

[20] Del Carpio C.A., Takahashi Y., and Sasaki S., A new approach to the automatic identification of candidates for ligand receptor sites in proteins: (I). Search for pocket regions. J Mol Graph, 1993 11(1): p. 23–9, 42 8499393 10.1016/0263-7855(93)85003-9

[21] Masuya M. and Doi J., Detection and geometric modeling of molecular surfaces and cavities using digital mathematical morphological operations. J Mol Graph, 1995 13(6): p. 331–6. 8820301 10.1016/0263-7855(95)00071-2

[22] Delaney J.S., Finding and filling protein cavities using cellular logic operations. J Mol Graph, 1992 10(3): p. 174–7, 163 1467333 10.1016/0263-7855(92)80052-f

[23] Kozlikova B., et al, CAVER Analyst 1.0: graphic tool for interactive visualization and analysis of tunnels and channels in protein structures. Bioinformatics, 2014 30(18): p. 2684–5. 10.1093/bioinformatics/btu364 24876375

[24] Chovancova E., et al, CAVER 3.0: a tool for the analysis of transport pathways in dynamic protein structures. PLoS Comput Biol, 2012 8(10): p. e1002708 10.1371/journal.pcbi.1002708 23093919 PMC3475669

[25] Yaffe E., et al, MolAxis: efficient and accurate identification of channels in macromolecules. Proteins, 2008 73(1): p. 72–86. 10.1002/prot.22052 18393395 PMC2693897

[26] Brylinski M., et al, Prediction of functional sites based on the fuzzy oil drop model. PLoS Comput Biol, 2007 3(5): p. e94 10.1371/journal.pcbi.0030094 17530916 PMC1876487

[27] Elcock A.H., Prediction of functionally important residues based solely on the computed energetics of protein structure. J Mol Biol, 2001 312(4): p. 885–96. 10.1006/jmbi.2001.5009 11575940

[28] Ondrechen M.J., Clifton J.G., and Ringe D., THEMATICS: a simple computational predictor of enzyme function from structure. Proc Natl Acad Sci U S A, 2001 98(22): p. 12473–8. 10.1073/pnas.211436698 11606719 PMC60078

[29] Goodford P.J., A computational procedure for determining energetically favorable binding sites on biologically important macromolecules. J Med Chem, 1985 28(7): p. 849–57. 3892003 10.1021/jm00145a002

[30] Ruppert J., Welch W., and Jain A.N., Automatic identification and representation of protein binding sites for molecular docking. Protein Sci, 1997 6(3): p. 524–33. 10.1002/pro.5560060302 9070435 PMC2143670

[31] Ghersi D. and Sanchez R., EasyMIFS and SiteHound: a toolkit for the identification of ligand-binding sites in protein structures. Bioinformatics, 2009 25(23): p. 3185–6. 10.1093/bioinformatics/btp562 19789268 PMC2913663

[32] Ben-Shimon A. and Eisenstein M., Looking at enzymes from the inside out: the proximity of catalytic residues to the molecular centroid can be used for detection of active sites and enzyme-ligand interfaces. J Mol Biol, 2005 351(2): p. 309–26. 10.1016/j.jmb.2005.06.047 16019028

[33] Gutteridge A., Bartlett G.J., and Thornton J.M., Using a neural network and spatial clustering to predict the location of active sites in enzymes. J Mol Biol, 2003 330(4): p. 719–34. 12850142 10.1016/s0022-2836(03)00515-1

[34] Petrova N.V. and Wu C.H., Prediction of catalytic residues using Support Vector Machine with selected protein sequence and structural properties. BMC Bioinformatics, 2006 7: p. 312 10.1186/1471-2105-7-312 16790052 PMC1534064

[35] Capra J.A., et al, Predicting protein ligand binding sites by combining evolutionary sequence conservation and 3D structure. PLoS Comput Biol, 2009 5(12): p. e1000585 10.1371/journal.pcbi.1000585 19997483 PMC2777313

[36] Dundas J., et al, CASTp: computed atlas of surface topography of proteins with structural and topographical mapping of functionally annotated residues. Nucleic Acids Res, 2006 34(Web Server issue): p. W116–8. 10.1093/nar/gkl282 16844972 PMC1538779

[37] Golovin A. and Henrick K., MSDmotif: exploring protein sites and motifs. BMC Bioinformatics, 2008 9: p. 312 10.1186/1471-2105-9-312 18637174 PMC2491636

[38] Shatsky M., et al, BioInfo3D: a suite of tools for structural bioinformatics. Nucleic Acids Res, 2004 32(Web Server issue): p. W503–7. 10.1093/nar/gkh413 15215437 PMC441551

[39] Tan K.P., et al, Depth: a web server to compute depth, cavity sizes, detect potential small-molecule ligand-binding cavities and predict the pKa of ionizable residues in proteins. Nucleic Acids Res, 2013 41(Web Server issue): p. W314–21. 10.1093/nar/gkt503 23766289 PMC3692129

[40] Kufareva I., Ilatovskiy A.V., and Abagyan R., Pocketome: an encyclopedia of small-molecule binding sites in 4D. Nucleic Acids Res, 2012 40(Database issue): p. D535–40. 10.1093/nar/gkr825 22080553 PMC3245087

[41] Roche D.B., Tetchner S.J., and McGuffin L.J., FunFOLD: an improved automated method for the prediction of ligand binding residues using 3D models of proteins. BMC Bioinformatics, 2011 12: p. 160 10.1186/1471-2105-12-160 21575183 PMC3123233

[42] Kellenberger E., et al, sc-PDB: an annotated database of druggable binding sites from the Protein Data Bank. J Chem Inf Model, 2006 46(2): p. 717–27. 10.1021/ci050372x 16563002

[43] Shoemaker B.A., et al, IBIS (Inferred Biomolecular Interaction Server) reports, predicts and integrates multiple types of conserved interactions for proteins. Nucleic Acids Res, 2012 40(Database issue): p. D834–40. 10.1093/nar/gkr997 22102591 PMC3245142

[44] Shulman-Peleg A., et al, MultiBind and MAPPIS: webservers for multiple alignment of protein 3D-binding sites and their interactions. Nucleic Acids Res, 2008 36(Web Server issue): p. W260–4. 10.1093/nar/gkn185 18467424 PMC2447750

[45] Tseng Y.Y., Chen Z.J., and Li W.H., fPOP: footprinting functional pockets of proteins by comparative spatial patterns. Nucleic Acids Res, 2010 38(Database issue): p. D288–95. 10.1093/nar/gkp900 19880384 PMC2808891

[46] Skolnick J. and Brylinski M., FINDSITE: a combined evolution/structure-based approach to protein function prediction. Brief Bioinform, 2009 10(4): p. 378–91. 10.1093/bib/bbp017 19324930 PMC2691936

[47] Brooks B. and Karplus M., Harmonic dynamics of proteins: normal modes and fluctuations in bovine pancreatic trypsin inhibitor. Proc Natl Acad Sci U S A, 1983 80(21): p. 6571–6575. 6579545 10.1073/pnas.80.21.6571 PMC391211

[48] Go N., Noguti T., and Nishikawa T., Dynamics of a small globular protein in terms of low-frequency vibrational modes. Proc Natl Acad Sci U S A, 1983 80(12): p. 3696–3700. 6574507 10.1073/pnas.80.12.3696 PMC394117

[49] Levitt M., Sander C., and Stern P.S., Protein normal-mode dynamics: trypsin inhibitor, crambin, ribonuclease and lysozyme. J Mol Biol, 1985 181(3): p. 423–447. 2580101 10.1016/0022-2836(85)90230-x

[50] Tirion M.M., Large Amplitude Elastic Motions in Proteins from a Single-Parameter, Atomic Analysis. Phys Rev Lett, 1996 77(9): p. 1905–1908. 10.1103/PhysRevLett.77.1905 10063201

[51] Delarue M. and Sanejouand Y.H., Simplified normal mode analysis of conformational transitions in DNA-dependent polymerases: the elastic network model. J Mol Biol, 2002 320(5): p. 1011–1024. 12126621 10.1016/s0022-2836(02)00562-4

[52] Suhre K. and Sanejouand Y.H., ElNemo: a normal mode web server for protein movement analysis and the generation of templates for molecular replacement. Nucleic Acids Res, 2004 32(Web Server issue): p. W610–4. 10.1093/nar/gkh368 15215461 PMC441506

[53] Van Der Spoel D., et al, GROMACS: fast, flexible, and free. J Comput Chem, 2005 26(16): p. 1701–1718. 10.1002/jcc.20291 16211538

[54] Samson A.O. and Levitt M., Inhibition mechanism of the acetylcholine receptor by alpha-neurotoxins as revealed by normal-mode dynamics. Biochemistry, 2008 47(13): p. 4065–70. 10.1021/bi702272j 18327915 PMC2750825

[55] Bhinge A., et al, Accurate detection of protein:ligand binding sites using molecular dynamics simulations. Structure, 2004 12(11): p. 1989–99. 10.1016/j.str.2004.09.005 15530363

[56] Ming D. and Wall M.E., Interactions in native binding sites cause a large change in protein dynamics. J Mol Biol, 2006 358(1): p. 213–23. 10.1016/j.jmb.2006.01.097 16513135

[57] Weng Y.Z., et al, A study on the flexibility of enzyme active sites. BMC Bioinformatics, 2011 12 Suppl 1: p. S32. 21342563 10.1186/1471-2105-12-S1-S32 PMC3044288

[58] Babor M., et al, Flexibility of metal binding sites in proteins on a database scale. Proteins, 2005 59(2): p. 221–30. 10.1002/prot.20431 15726624

[59] Porter C.T., Bartlett G.J., and Thornton J.M., The Catalytic Site Atlas: a resource of catalytic sites and residues identified in enzymes using structural data. Nucleic Acids Res, 2004 32(Database issue): p. D129–33. 10.1093/nar/gkh028 14681376 PMC308762

[60] Kabsch W. and Sander C., Dictionary of protein secondary structure: pattern recognition of hydrogen-bonded and geometrical features. Biopolymers, 1983 22(12): p. 2577–637. 10.1002/bip.360221211 6667333

[61] Huang B. and Schroeder M., LIGSITEcsc: predicting ligand binding sites using the Connolly surface and degree of conservation. BMC Struct Biol, 2006 6: p. 19 10.1186/1472-6807-6-19 16995956 PMC1601958

[62] Puvanendrampillai D. and Mitchell J.B., L/D Protein Ligand Database (PLD): additional understanding of the nature and specificity of protein-ligand complexes. Bioinformatics, 2003 19(14): p. 1856–7. 14512362 10.1093/bioinformatics/btg243

[63] Huang B., MetaPocket: a meta approach to improve protein ligand binding site prediction. OMICS, 2009 13(4): p. 325–30. 10.1089/omi.2009.0045 19645590

[64] DesJarlais R.L., et al, Using shape complementarity as an initial screen in designing ligands for a receptor binding site of known three-dimensional structure. J Med Chem, 1988 31(4): p. 722–9. 3127588 10.1021/jm00399a006

[65] Levitt M., et al, Potential energy function and parameters for simulations of the molecular dynamics of proteins and nucleic acids in solution. Computer Physics Communications, 1995 91: p. 215–231.

