# Supplementary figures and images for: Normal Modes Expose Active Sites in Enzymes
Source: PLoS Comput Biol. 2016 Dec 21;12(12):e1005293. doi: 10.1371/journal.pcbi.1005293 (PMC5225006; doi:10.1371/journal.pcbi.1005293)

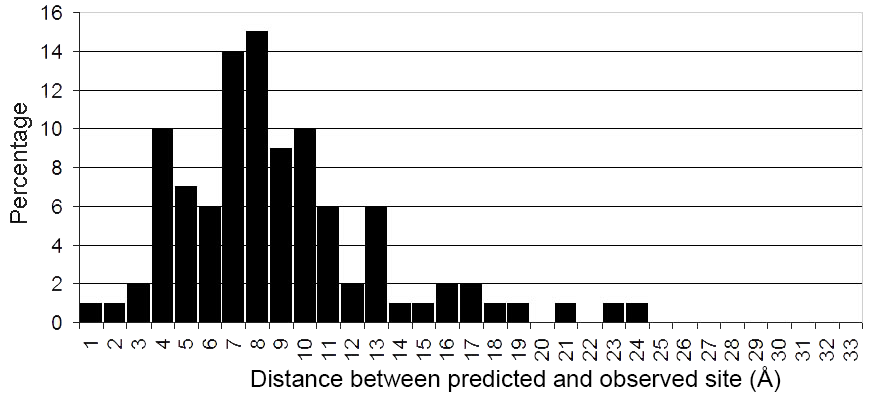

Supplement: S1 Fig — The distribution of distances between the predicted and observed active sites is shown. Note that 92% of the predictions fall within 12 Å of the observed active site. (TIF) [file pcbi.1005293.s001.tif]

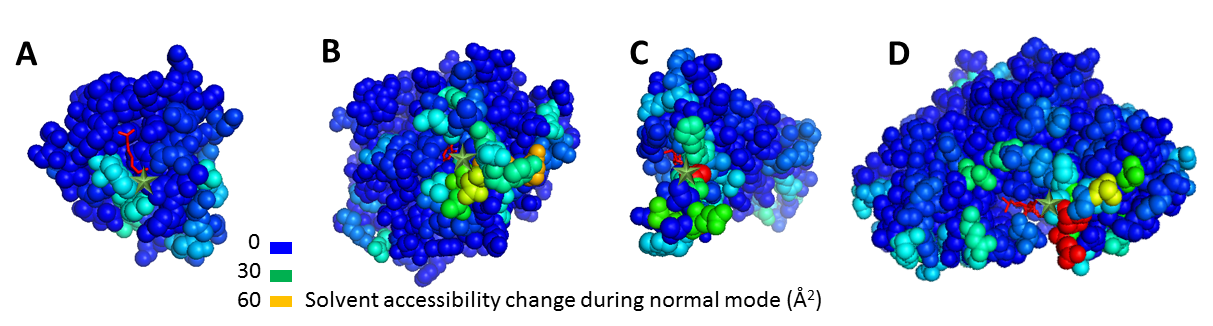

Supplement: S2 Fig — Shown are four additional EXPOSITE predictions for the enzymes (A) 2pk4, (B) 1ulb, (C) 1stp, and (D) 1apu of the PLD dataset. The predicted and observed binding sites are indicated by green stars and red ligands respectively, and LIGSITE pockets are displayed as white spheres. In cyan and green, are residues displaying large changes of accessibility in normal modes, and in blue, are residues which display little or no change of exposure. Note that the ligand (in red) is within 4Å of the predicted site (green star). The figure was prepared using Pymol. (TIF) [file pcbi.1005293.s002.tif]
