# Supplementary material for: Normal Modes Expose Active Sites in Enzymes
Source: PLoS Comput Biol. 2016 Dec 21;12(12):e1005293. doi: 10.1371/journal.pcbi.1005293 (PMC5225006; doi:10.1371/journal.pcbi.1005293)
Supplement: S1 Table — (DOCX) [file pcbi.1005293.s003.docx]

***Supplementary table 1.*** List of 133 enzyme training dataset.

_________________________________________________

132l 135l 1a0i 1a26 1a2t 1a65 1a8h 1af7 1ah7 1aj0 1ak0 1akd 1ako 1amy 1aop 1aq2 1ast 1auk 1b6g 1bg0 1bh2 1bib 1bob 1boo 1bp2 1bqc 1bs9 1bsj 1btl 1bvv 1bwz 1bya 1c82 1ca3 1coy 1cqq 1cv2 1cwy 1din 1dj1 1dl2 1dve 1e0c 1eb6 1eh5 1eh6 1eo7 1eug 1ex1 1exp 1fgh 1fhl 1fnb 1foa 1fob 1fy2 1g6t 1g8o 1g8p 1gal 1gcu 1glo 1gns 1gq8 1h19 1hka 1hpm 1i1i 1ig8 1it4 1j00 1j53 1jms 1k30 1kaz 1knp 1kzl 1l6p 1l7q 1l8t 1lba 1lbu 1lci 1lij 1lio 1lml 1lz1 1mbb 1mj9 1mla 1mrq 1mud 1mug 1n29 1ndh 1nml 1og1 1ogo 1oh9 1opm 1oxa 1p5d 1pgs 1pja 1pkn 1pmi 1ps9 1q91 1qaz 1qba 1qe3 1qfm 1qje 1qv0 1ra2 1rbn 1rhs 1rtu 1ru4 1sca 1sll 1ssx 1uas 1uch 1v0y 1vid 1vnc 1w0h 1w1o 1y9m 1yon 1ytw 1zio
