# Supplementary material for: Normal Modes Expose Active Sites in Enzymes
Source: PLoS Comput Biol. 2016 Dec 21;12(12):e1005293. doi: 10.1371/journal.pcbi.1005293 (PMC5225006; doi:10.1371/journal.pcbi.1005293)
Supplement: S2 Table — (DOCX) [file pcbi.1005293.s004.docx]

***Supplementary table 2.*** List of 845 enzyme testing dataset.

12as 13pk 1a05 1a0j 1a16 1a30 1a41 1a4g 1a4i 1a4l 1a4s 1a69 1a6d 1a79 1a7u 1a8q 1a8s 1a95 1aa6 1aam 1ab4 1abr 1adn 1afr 1afw 1agm 1agy 1ahj 1aj8 1akm 1al6 1ald 1alk 1am2 1am5 1amo 1amp 1apx 1apy 1aq0 1aql 1ar1 1arz 1at1 1aug 1aui 1auo 1auw 1avf 1ax4 1ay4 1azw 1b02 1b04 1b2m 1b2r 1b3m 1b3r 1b57 1b5d 1b5q 1b5t 1b65 1b66 1b6b 1b6t 1b73 1b7y 1b8b 1b8f 1b8g 1b93 1b9h 1bd0 1bd3 1be1 1bf2 1bfd 1bg6 1bgl 1bhg 1bix 1bjo 1bjp 1bmt 1bo1 1bol 1brm 1brw 1bs0 1bs4 1bt1 1bu7 1bvz 1bwd 1bwl 1b9h 1bd0 1bd3 1be1 1bf2 1bfd 1bg6 1bgl 1bhg 1bix 1bjo 1bjp 1bwp 1bzc 1bzy 1c17 1c2t 1c3c 1c3j 1c4t 1c4x 1c4z 1c54 1c9u 1ca2 1cb8 1cbg 1cbx 1cd5 1cde 1cdg 1cel 1cev 1cfr 1cg2 1cg6 1cgk 1chd 1chk 1chm 1ci8 1cjy 1ck7 1cl1 1cm0 1cms 1cmx 1cns 1cqg 1cqj 1cs1 1ct9 1ctn 1ctt 1cvr 1cw0 1cz0 1cz1 1czf 1d0s 1d1q 1d2h 1d2r 1d2t 1d3g 1d4a 1d4c 1d5r 1d6i 1d6m 1d6o 1d7r 1d8c 1d8d 1d8h 1d8t 1daa 1dae 1db3 1dbf 1dbt 1dci 1dco 1dd8 1ddj 1de3 1dek 1dfo 1dgs 1dhf 1dhp 1dhr 1di1 1dii 1dio 1diz 1dj0 1djl 1djo 1dki 1dl5 1dli 1dmu 1dnk 1dnp 1do8 1dod 1doo 1ddj 1de3 1dek 1dfo 1dgs 1dhf 1dhp 1dhr 1di1 1dii 1dio 1diz 1dpg 1dqa 1dqr 1dqs 1dtw 1dub 1dup 1dw9 1dxe 1dzr 1e19 1e1a 1e2a 1e2t 1e3v 1e5q 1e6e 1e7l 1e7q 1eag 1ebf 1ec9 1ecf 1ecl 1ecm 1ecx 1eej 1ef0 1ef8 1eg7 1ehk 1ehy 1ei5 1eix 1elq 1els 1emd 1eq2 1esc 1eso 1et0 1eu1 1eul 1euu 1euy 1evy 1exn 1ey2 1eyi 1eyp 1ez1 1ez2 1f2d 1f48 1f6d 1f75 1f7l 1f7u 1f80 1f8m 1f8r 1f8x 1fa0 1fcb 1fcq 1fdy 1ff3 1fft 1fgj 1fo6 1fps 1fq0 1fr8 1fro 1fsg 1fua 1fug 1fui 1fuq 1fva 1fwk 1g0d 1g24 1g4p 1g64 1g72 1g79 1g8f 1g99 1ga8 1gcb 1gdh 1gdo 1ge7 1geq 1get 1ghs 1gim 1gog 1goj 1gox 1gp1 1gp5 1gpa 1gpj 1gpm 1gpr 1gqg 1g64 1g72 1g79 1g8f 1g99 1ga8 1gcb 1gdh 1gdo 1ge7 1geq 1get 1grc 1gsa 1gt7 1gtp 1gtx 1guf 1gxs 1gz6 1h3i 1h4g 1h54 1h7o 1h7x 1hdh 1hfs 1hiv 1hpl 1hqc 1hr6 1hr7 1hrd 1hrk 1hti 1hto 1hxq 1hy3 1hzd 1hzf 1i1e 1i29 1i6p 1i78 1i7q 1i8d 1i8t 1i9a 1idj 1idt 1iec 1im5 1ima 1inp 1iph 1itq 1itx 1iu4 1ivh 1iyd 1j09 1j2u 1j49 1j70 1j79 1j7g 1jag 1jch 1jdw 1jen 1jfl 1jh6 1jhf 1jkm 1jm6 1jnr 1joa 1jof 1jqn 1js4 1jxa 1jxh 1k0w 1k32 1k4l 1k4t 1k82 1kae 1kas 1kc7 1kcz 1kdg 1kez 1kim 1kl7 1kny 1kp2 1kqc 1kqf 1ksj 1kws 1kyq 1kyw 1kzh 1l0o 1l1d 1l1l 1l1r 1l7d 1l7n 1l9x 1lam 1lcb 1ldm 1lj1 1ljl 1lnh 1ltq 1luc 1lvh 1kp2 1kqc 1kqf 1ksj 1kws 1kyq 1kyw 1kzh 1l0o 1l1d 1l1l 1l1r 1lws 1lxa 1lya 1m21 1m53 1m54 1m6k 1m9c 1mas 1mek 1mfp 1mhl 1mht 1mka 1mlv 1mok 1moq 1mpx 1mpy 1mqw 1mro 1mt5 1muc 1mvn 1myr 1n20 1n2c 1n2t 1naa 1nba 1nbf 1ndi 1ndo 1nf9 1nhx 1ni4 1nid 1nir 1nkk 1nln 1nlu 1nmw 1nsf 1nsj 1nsp 1nu3 1nvm 1nvt 1nww 1nzy 1o04 1o8a 1o98 1o9i 1oac 1oas 1oba 1oe8 1ofd 1ofg 1ohh 1oj4 1ok4 1okg 1onr 1oqz 1or8 1ord 1oro 1os7 1otg 1oya 1oyg 1ozh 1p1x 1p3d 1p4n 1p4r 1p7m 1pa9 1pad 1pbg 1peg 1pfk 1pfq 1pii 1pix 1pj5 1pjb 1pjh 1pjq 1pma 1pnt 1pow 1pp4 1ps1 1psd 1ptd 1pud 1pvd 1pvi 1pwh 1pwv 1pxv 1pya 1pyl 1pym 1pz3 1pfq 1pii 1pix 1pj5 1pjb 1pjh 1pjq 1pma 1pnt 1pow 1pp4 1ps1 1q18 1q3n 1q3q 1q6l 1q6x 1qam 1qb4 1qcn 1qd1 1qf6 1qfe 1qfl 1qfn 1qgn 1qgx 1qh5 1qh9 1qhf 1qhg 1qho 1qi9 1qib 1qj4 1qk2 1qlh 1qmh 1qol 1qpr 1qq5 1qrg 1qrr 1qrz 1qsg 1qtn 1qum 1qwn 1qx3 1qz9 1r16 1r1j 1r30 1r44 1r4f 1r4z 1r76 1ra0 1rba 1rbl 1rdd 1req 1rgq 1rhc 1rk2 1ro7 1roz 1rpt 1rpx 1rql 1rvv 1s20 1s2k 1s3i 1s95 1s9c 1ses 1slm 1sme 1sml 1smn 1snn 1sox 1std 1t0u 1t4c 1t7d 1tah 1tde 1tdj 1teh 1thg 1tht 1ti6 1tml 1tmo 1tox 1trk 1tyf 1tys 1tz3 1u3f 1u5u 1u7u 1u8v 1uae 1uag 1uaq 1uf7 1ujn 1uk7 1ula 1un1 1uok 1uqr 1uqt 1uro 1ush 1uw8 1v04 1tox 1trk 1tyf 1tys 1tz3 1u3f 1u5u 1u7u 1u8v 1uae 1uag 1uaq 1v0e 1v25 1vao 1vas 1vie 1vlb 1vom 1vq1 1vr7 1vzx 1vzz 1w27 1w2n 1wd8 1wgi 1wnw 1x7d 1x9h 1x9y 1xa8 1xgm 1xik 1xny 1xqd 1xqw 1xs1 1xtc 1xva 1xvt 1xyz 1ybq 1ybv 1ycf 1ygh 1ylu 1ysc 1z9h 1ze1 1zoi 1zrz 1zym 206l 2a0n 2a86 2aat 2abk 2ace 2acu 2acy 2adm 2ag0 2ahj 2alr 2amg 2apr 2ayh 2b3i 2bhg 2bif 2bkr 2blt 2bmi 2bsx 2bx4 2c7v 2cnd 2cpo 2cpu 2dbt 2dhn 2dln 2dor 2dw7 2ebn 2eng 2eql 2esd 2f61 2f9r 2fmn 2fok 2fqq 2gsa 2hdh 2hgs 2his 2hsa 2isd 2jcw 2jxr 2lip 2lpr 2nac 2nlr 2nmt 2npx 2dw7 2ebn 2eng 2eql 2esd 2f61 2f9r 2fmn 2fok 2fqq 2gsa 2hdh 2oat 2pda 2pec 2pfl 2pgd 2phk 2pia 2plc 2pth 2qf7 2rnf 2sqc 2tdt 2thi 2tmd 2toh 2tpl 2tps 2ts1 2xis 2ypn 3cla 3csm 3eca 3mdd 3nos 3pva 3r1r 4kbp 4mdh 5cox 5cpa 5eat 5enl 5fit 5rsa 7atj 7nn9 7odc 8pch 9pap
