# Supplementary material for: Normal Modes Expose Active Sites in Enzymes
Source: PLoS Comput Biol. 2016 Dec 21;12(12):e1005293. doi: 10.1371/journal.pcbi.1005293 (PMC5225006; doi:10.1371/journal.pcbi.1005293)
Supplement: S3 Table — (DOCX) [file pcbi.1005293.s005.docx]

***Supplementary table 3.*** List of 48 proteins dataset derived from the Protein Ligand Database (PLD) by Huang et al.

1bid 1cdo 1dwd 1fbp 1gca 1hew 1hyt 1inc 1rbp 1rob 1stp 1ulb 1ifb 3ptb 2ypi 4dfr 4phv 5cna 7cpa 1a6w 1apu 1acj 1blh 1byb 1hfc 1ida 1ivd 1mrg 1mtw 1okm 1pdz 1phd 1pso 1qpe 1rne 1snc 1srf 1stp 2ctc 2h4n 2pk4 2sim 2tmn 3gch 3mth 5p2p 1imb 6rsa
