# Supplementary material for: Normal Modes Expose Active Sites in Enzymes
Source: PLoS Comput Biol. 2016 Dec 21;12(12):e1005293. doi: 10.1371/journal.pcbi.1005293 (PMC5225006; doi:10.1371/journal.pcbi.1005293)
Supplement: S4 Table — (DOCX) [file pcbi.1005293.s006.docx]

***Supplementary table 4.*** Training of EXPOSITE using different solvent accessible cutoffs in the 133 enzyme test dataset

| **\|ΔSAS\|_max_ cutoffs (Å^2^)** | **\|ΔSAS\|_min_ cutoffs (Å^2^)** | | | | | |
| --- | --- | --- | --- | --- | --- | --- |
|  | **0** | **10** | **20** | **30** | **40** | **50** |
| **10** | 86 |  |  |  |  |  |
| **20** | 86 | 88 |  |  |  |  |
| **30** | 86 | 89 | 89 |  |  |  |
| **40** | 86 | 89 | 92 | 89 |  |  |
| **50** | 86 | 89 | 91 | 88 | 81 |  |
| **60** | 86 | 90 | 91 | 88 | 88 | 86 |
