# Supplementary material for: Normal Modes Expose Active Sites in Enzymes
Source: PLoS Comput Biol. 2016 Dec 21;12(12):e1005293. doi: 10.1371/journal.pcbi.1005293 (PMC5225006; doi:10.1371/journal.pcbi.1005293)
Supplement: S5 Table — (DOCX) [file pcbi.1005293.s007.docx]

***Supplementary table 5.*** Training of EXPOSITE using different numbers of modes in the 133 enzyme test dataset.

| **No. of modes sampled** | **Success rate (%)** |
| --- | --- |
| **1** | 83 |
| **2** | 84 |
| **3** | 86 |
| **4** | 86 |
| **5** | 86 |
| **6** | 87 |
| **7** | 89 |
| **8** | 92 |
| **9** | 92 |
| **10** | 92 |
