# Supplementary material for: Normal Modes Expose Active Sites in Enzymes
Source: PLoS Comput Biol. 2016 Dec 21;12(12):e1005293. doi: 10.1371/journal.pcbi.1005293 (PMC5225006; doi:10.1371/journal.pcbi.1005293)
Supplement: S6 Table — (DOCX) [file pcbi.1005293.s008.docx]

***Supplementary table 6.*** List of success and failures of EXPOSITE in the 133 enzyme dataset

| PDB ID | Success | Pocket no. |
| --- | --- | --- |
| 132l | yes | 1 |
| 135l | yes | 1 |
| 1a0i | yes | 6 |
| 1a26 | yes | 1 |
| 1a2t | yes | 1 |
| 1a65 | yes | 8 |
| 1a8h | yes | 1 |
| 1af7 | yes | 1 |
| 1ah7 | yes | 1 |
| 1aj0 | yes | 1 |
| 1ak0 | yes | 1 |
| 1akd | yes | 1 |
| 1ako | yes | 3 |
| 1amy | yes | 1 |
| 1aop | yes | 1 |
| 1aq2 | yes | 1 |
| 1ast | yes | 1 |
| 1auk | yes | 1 |
| 1b6g | No |  |
| 1bg0 | yes | 1 |
| 1bh2 | yes | 1 |
| 1bib | yes | 1 |
| 1bob | yes | 1 |
| 1boo | yes | 1 |
| 1bp2 | yes | 1 |
| 1bqc | yes | 1 |
| 1bs9 | yes | 2 |
| 1bsj | yes | 1 |
| 1btl | yes | 2 |
| 1bvv | yes | 1 |
| 1bwz | yes | 1 |
| 1bya | yes | 1 |
| 1c82 | yes | 1 |
| 1ca3 | yes | 1 |
| 1coy | yes | 1 |
| 1cqq | yes | 1 |
| 1cv2 | yes | 1 |
| 1cwy | yes | 1 |
| 1din | yes | 1 |
| 1dj1 | yes | 1 |
| 1dl2 | yes | 1 |
| 1dve | yes | 1 |
| 1e0c | yes | 7 |
| 1eb6 | yes | 1 |
| 1eh5 | yes | 1 |
| 1eh6 | yes | 2 |
| 1eo7 | No |  |
| 1eug | yes | 1 |
| 1ex1 | No |  |
| 1exp | yes | 1 |
| 1fgh | yes | 1 |
| 1fhl | yes | 1 |
| 1fnb | yes | 1 |
| 1foa | yes | 1 |
| 1fob | yes | 1 |
| 1fy2 | yes | 1 |
| 1g6t | yes | 1 |
| 1g8o | yes | 1 |
| 1g8p | No |  |
| 1gal | yes | 1 |
| 1gcu | yes | 1 |
| 1glo | yes | 2 |
| 1gns | yes | 1 |
| 1gq8 | yes | 2 |
| 1h19 | yes | 1 |
| 1hka | yes | 1 |
| 1hpm | yes | 1 |
| 1i1i | yes | 1 |
| 1ig8 | yes | 1 |
| 1it4 | yes | 1 |
| 1j00 | yes | 1 |
| 1j53 | yes | 1 |
| 1jms | yes | 1 |
| 1k30 | yes | 1 |
| 1kaz | yes | 1 |
| 1knp | yes | 1 |
| 1kzl | yes | 4 |
| 1l6p | yes | 1 |
| 1l7q | yes | 1 |
| 1l8t | yes | 1 |
| 1lba | yes | 2 |
| 1lbu | No |  |
| 1lci | yes | 1 |
| 1lij | yes | 1 |
| 1lio | yes | 1 |
| 1lml | yes | 1 |
| 1lz1 | yes | 1 |
| 1mbb | yes | 1 |
| 1mj9 | yes | 1 |
| 1mla | yes | 1 |
| 1mrq | yes | 1 |
| 1mud | yes | 1 |
| 1mug | yes | 1 |
| 1n29 | yes | 1 |
| 1ndh | yes | 1 |
| 1nml | yes | 1 |
| 1og1 | yes | 1 |
| 1ogo | yes | 1 |
| 1oh9 | yes | 1 |
| 1opm | yes | 1 |
| 1oxa | yes | 1 |
| 1p5d | yes | 1 |
| 1pgs | yes | 1 |
| 1pja | yes | 1 |
| 1pkn | yes | 1 |
| 1pmi | yes | 1 |
| 1ps9 | No |  |
| 1q91 | yes | 1 |
| 1qaz | yes | 1 |
| 1qba | yes | 4 |
| 1qe3 | No |  |
| 1qfm | yes | 1 |
| 1qje | yes | 1 |
| 1qv0 | yes | 1 |
| 1ra2 | yes | 1 |
| 1rbn | yes | 1 |
| 1rhs | No |  |
| 1rtu | No |  |
| 1ru4 | yes | 2 |
| 1sca | yes | 4 |
| 1sll | yes | 3 |
| 1ssx | yes | 1 |
| 1uas | yes | 1 |
| 1uch | No |  |
| 1v0y | Yes | 1 |
| 1vid | Yes | 1 |
| 1vnc | Yes | 1 |
| 1w0h | Yes | 1 |
| 1w1o | Yes | 1 |
| 1y9m | No |  |
| 1yon | Yes | 1 |
| 1ytw | Yes | 7 |
| 1zio | Yes | 1 |
