# Supplementary material for: Normal Modes Expose Active Sites in Enzymes
Source: PLoS Comput Biol. 2016 Dec 21;12(12):e1005293. doi: 10.1371/journal.pcbi.1005293 (PMC5225006; doi:10.1371/journal.pcbi.1005293)
Supplement: S7 Table — (DOCX) [file pcbi.1005293.s009.docx]

***Supplementary table 7.*** List of success and failures of EXPOSITE in the 845 enzyme dataset.

| PDB ID | Success  EXPOSITE | Pocket no. |
| --- | --- | --- |
| 12as | Yes | 1 |
| 135l | Yes | 1 |
| 13pk | Yes | 1 |
| 1a05 | Yes | 2 |
| 1a0i | Yes | 1 |
| 1a0j | Yes | 3 |
| 1a16 | Yes | 4 |
| 1a26 | Yes | 2 |
| 1a2t | Yes | 1 |
| 1a30 | No |  |
| 1a41 | yes | 1 |
| 1a4g | yes | 1 |
| 1a4i | yes | 1 |
| 1a4s | yes | 1 |
| 1a65 | yes | 8 |
| 1a6d | no |  |
| 1a79 | yes | 1 |
| 1a7u | no |  |
| 1a8q | yes | 1 |
| 1a8s | yes | 1 |
| 1a95 | yes | 1 |
| 1aa6 | no |  |
| 1aam | yes | 1 |
| 1ab4 | yes | 2 |
| 1abr | no |  |
| 1af7 | yes | 1 |
| 1afr | no |  |
| 1afw | yes | 1 |
| 1agm | no |  |
| 1ah7 | yes | 1 |
| 1ahj | yes | 1 |
| 1aj0 | yes | 1 |
| 1aj8 | no |  |
| 1ak0 | yes | 1 |
| 1akm | yes | 1 |
| 1ako | yes | 3 |
| 1al6 | yes | 1 |
| 1ald | yes | 1 |
| 1alk | yes | 1 |
| 1am2 | no |  |
| 1am5 | yes | 1 |
| 1amo | yes | 1 |
| 1amp | yes | 1 |
| 1amy | yes | 1 |
| 1aop | yes | 1 |
| 1apx | yes | 1 |
| 1apy | yes | 1 |
| 1aq0 | yes | 3 |
| 1aq2 | yes | 1 |
| 1aql | yes | 1 |
| 1ar1 | yes | 1 |
| 1arz | yes | 1 |
| 1ast | yes | 1 |
| 1at1 | yes | 1 |
| 1aug | yes | 1 |
| 1aui | yes | 3 |
| 1auk | yes | 1 |
| 1auo | yes | 1 |
| 1auw | no |  |
| 1avf | yes | 1 |
| 1ax4 | yes | 1 |
| 1ay4 | yes | 1 |
| 1azw | yes | 1 |
| 1b02 | yes | 1 |
| 1b04 | no |  |
| 1b2m | no |  |
| 1b2r | yes | 1 |
| 1b3r | yes | 1 |
| 1b57 | yes | 1 |
| 1b5d | yes | 1 |
| 1b5q | yes | 1 |
| 1b5t | yes | 1 |
| 1b65 | yes | 1 |
| 1b66 | yes | 1 |
| 1b6b | yes | 1 |
| 1b6g | no |  |
| 1b6t | yes | 1 |
| 1b73 | yes | 1 |
| 1b7y | yes | 1 |
| 1b8f | yes | 1 |
| 1b8g | yes | 1 |
| 1b93 | yes | 1 |
| 1b9h | yes | 1 |
| 1bd0 | no |  |
| 1bd3 | yes | 1 |
| 1be1 | no |  |
| 1bf2 | no |  |
| 1bfd | yes | 3 |
| 1bg0 | yes | 1 |
| 1bg6 | yes | 1 |
| 1bgl | yes | 2 |
| 1bh2 | yes | 1 |
| 1bhg | no |  |
| 1bib | yes | 1 |
| 1bix | yes | 1 |
| 1bjo | yes | 2 |
| 1bjp | no |  |
| 1bmt | yes | 1 |
| 1bo1 | yes | 1 |
| 1bob | yes | 1 |
| 1bol | no |  |
| 1boo | yes | 1 |
| 1bp2 | yes | 1 |
| 1bqc | no |  |
| 1brm | yes | 2 |
| 1brw | yes | 1 |
| 1bs0 | yes | 1 |
| 1bs4 | yes | 1 |
| 1bs9 | no |  |
| 1bsj | yes | 1 |
| 1bt1 | yes | 1 |
| 1btl | yes | 2 |
| 1bu7 | yes | 1 |
| 1bvz | no |  |
| 1bwd | yes | 1 |
| 1bwl | yes | 1 |
| 1bwp | yes | 2 |
| 1bwz | no |  |
| 1bya | yes | 1 |
| 1bzc | yes | 1 |
| 1bzy | yes | 1 |
| 1c17 | no |  |
| 1c2t | yes | 1 |
| 1c3c | no |  |
| 1c3j | yes | 1 |
| 1c4t | yes | 1 |
| 1c4x | yes | 1 |
| 1c4z | no |  |
| 1c82 | yes | 5 |
| 1c9u | yes | 1 |
| 1ca2 | yes | 1 |
| 1ca3 | yes | 1 |
| 1cb8 | yes | 1 |
| 1cbg | yes | 1 |
| 1cbx | yes | 1 |
| 1cd5 | yes | 1 |
| 1cde | yes | 1 |
| 1cdg | no |  |
| 1cel | yes | 1 |
| 1cev | yes | 2 |
| 1cfr | yes | 1 |
| 1cg2 | yes | 1 |
| 1cg6 | no |  |
| 1cgk | yes | 1 |
| 1chd | yes | 1 |
| 1chk | yes | 1 |
| 1chm | yes | 1 |
| 1ci8 | yes | 1 |
| 1cjy | no |  |
| 1ck7 | no |  |
| 1cl1 | yes | 1 |
| 1cm0 | yes | 1 |
| 1cms | yes | 1 |
| 1cmx | yes | 1 |
| 1cns | yes | 1 |
| 1coy | yes | 1 |
| 1cqj | yes | 1 |
| 1cqq | no |  |
| 1cs1 | yes | 1 |
| 1ct9 | yes | 3 |
| 1ctn | yes | 1 |
| 1ctt | yes | 1 |
| 1cv2 | yes | 1 |
| 1cvr | yes | 1 |
| 1cw0 | no |  |
| 1cwy | no |  |
| 1cz0 | no |  |
| 1cz1 | yes | 1 |
| 1czf | yes | 1 |
| 1d0s | yes | 1 |
| 1d1q | yes | 1 |
| 1d2h | yes | 1 |
| 1d2r | no |  |
| 1d2t | yes | 1 |
| 1d3g | no |  |
| 1d4a | yes | 1 |
| 1d4c | no |  |
| 1d5r | no |  |
| 1d6i | yes | 1 |
| 1d6m | no |  |
| 1d6o | yes | 1 |
| 1d7r | yes | 1 |
| 1d8c | no |  |
| 1d8d | no |  |
| 1d8h | no |  |
| 1d8t | yes | 1 |
| 1daa | yes | 1 |
| 1dae | yes | 1 |
| 1db3 | yes | 1 |
| 1dbf | yes | 1 |
| 1dbt | yes | 1 |
| 1dci | no |  |
| 1dco | no |  |
| 1dd8 | yes | 1 |
| 1ddj | yes | 1 |
| 1dek | yes | 1 |
| 1dfo | yes | 1 |
| 1dgs | no |  |
| 1dhf | yes | 1 |
| 1dhp | yes | 2 |
| 1dhr | yes | 1 |
| 1di1 | yes | 1 |
| 1dii | yes | 1 |
| 1din | no |  |
| 1dio | yes | 3 |
| 1diz | yes | 1 |
| 1dj0 | yes | 1 |
| 1djl | yes | 2 |
| 1dki | yes | 2 |
| 1dl2 | yes | 1 |
| 1dl5 | yes | 1 |
| 1dli | yes | 1 |
| 1dmu | yes | 1 |
| 1dnk | no |  |
| 1dnp | yes | 1 |
| 1do8 | yes | 5 |
| 1dod | yes | 1 |
| 1dpg | yes | 1 |
| 1dqa | no |  |
| 1dqr | no |  |
| 1dqs | yes | 1 |
| 1dtw | yes | 4 |
| 1dub | no |  |
| 1dup | yes | 1 |
| 1dve | yes | 1 |
| 1dw9 | yes | 1 |
| 1dxe | yes | 2 |
| 1dzr | yes | 1 |
| 1e0c | yes | 1 |
| 1e19 | yes | 1 |
| 1e1a | no |  |
| 1e2a | no |  |
| 1e2t | yes | 1 |
| 1e3v | yes | 1 |
| 1e5q | yes | 1 |
| 1e6e | yes | 1 |
| 1e7l | no |  |
| 1e7q | yes | 1 |
| 1eag | yes | 1 |
| 1eb6 | yes | 1 |
| 1ebf | yes | 1 |
| 1ec9 | yes | 1 |
| 1ecf | no |  |
| 1ecl | no |  |
| 1ecm | yes | 1 |
| 1ecx | yes | 1 |
| 1eej | yes | 1 |
| 1ef0 | no |  |
| 1ef8 | no |  |
| 1eh5 | yes | 1 |
| 1eh6 | yes | 1 |
| 1ehy | no |  |
| 1ei5 | yes | 1 |
| 1eix | yes | 1 |
| 1elq | yes | 3 |
| 1els | yes | 1 |
| 1emd | yes | 1 |
| 1eo7 | no |  |
| 1eq2 | yes | 1 |
| 1esc | yes | 1 |
| 1eso | no |  |
| 1et0 | yes | 1 |
| 1eu1 | no |  |
| 1eug | yes | 1 |
| 1euu | no |  |
| 1euy | no |  |
| 1evy | yes | 1 |
| 1ex1 | no |  |
| 1exn | yes | 1 |
| 1exp | yes | 1 |
| 1ey2 | no |  |
| 1eyi | yes | 2 |
| 1eyp | yes | 1 |
| 1ez1 | yes | 1 |
| 1ez2 | yes | 1 |
| 1f2d | yes | 1 |
| 1f48 | yes | 1 |
| 1f6d | yes | 1 |
| 1f75 | yes | 1 |
| 1f7l | no |  |
| 1f7u | no |  |
| 1f80 | yes | 1 |
| 1f8m | yes | 5 |
| 1f8r | yes | 1 |
| 1f8x | yes | 1 |
| 1fa0 | no |  |
| 1fcb | yes | 1 |
| 1fcq | yes | 3 |
| 1fdy | yes | 1 |
| 1ff3 | yes | 1 |
| 1fft | yes | 1 |
| 1fgh | yes | 4 |
| 1fgj | yes | 2 |
| 1fhl | yes | 1 |
| 1fnb | yes | 1 |
| 1fo6 | yes | 1 |
| 1foa | yes | 1 |
| 1fob | yes | 1 |
| 1fps | yes | 1 |
| 1fq0 | yes | 1 |
| 1fr8 | no |  |
| 1fro | yes | 1 |
| 1fsg | yes | 1 |
| 1fua | yes | 1 |
| 1fug | yes | 3 |
| 1fui | no |  |
| 1fuq | no |  |
| 1fva | yes | 1 |
| 1fwk | yes | 1 |
| 1fy2 | no |  |
| 1g0d | no |  |
| 1g24 | yes | 1 |
| 1g4p | yes | 1 |
| 1g64 | yes | 1 |
| 1g6t | yes | 1 |
| 1g72 | yes | 2 |
| 1g79 | yes | 1 |
| 1g8f | yes | 4 |
| 1g8o | yes | 1 |
| 1g8p | no |  |
| 1g99 | yes | 1 |
| 1ga8 | yes | 1 |
| 1gal | yes | 1 |
| 1gcb | yes | 1 |
| 1gcu | yes | 1 |
| 1gdh | yes | 1 |
| 1ge7 | yes | 1 |
| 1geq | yes | 1 |
| 1get | yes | 1 |
| 1ghs | yes | 1 |
| 1gim | no |  |
| 1glo | yes | 1 |
| 1gns | yes | 1 |
| 1gog | yes | 4 |
| 1goj | no |  |
| 1gox | no |  |
| 1gp1 | yes | 1 |
| 1gp5 | yes | 1 |
| 1gpa | yes | 1 |
| 1gpj | yes | 5 |
| 1gpm | no |  |
| 1gpr | yes | 1 |
| 1gq8 | no |  |
| 1gqg | no |  |
| 1grc | yes | 1 |
| 1gsa | yes | 1 |
| 1gt7 | no |  |
| 1gtp | yes | 1 |
| 1guf | yes | 1 |
| 1gxs | yes | 1 |
| 1gz6 | no |  |
| 1h19 | yes | 5 |
| 1h3i | no |  |
| 1h4g | yes | 1 |
| 1h54 | no |  |
| 1h7o | yes | 2 |
| 1hdh | no |  |
| 1hfs | yes | 1 |
| 1hiv | no |  |
| 1hka | yes | 1 |
| 1hpl | no |  |
| 1hpm | no |  |
| 1hqc | yes | 2 |
| 1hr6 | no |  |
| 1hr7 | no |  |
| 1hrd | yes | 1 |
| 1hrk | yes | 1 |
| 1hti | yes | 1 |
| 1hto | yes | 1 |
| 1hxq | no |  |
| 1hy3 | yes | 1 |
| 1hzd | no |  |
| 1hzf | no |  |
| 1i29 | yes | 1 |
| 1i6p | no |  |
| 1i78 | no |  |
| 1i7q | no |  |
| 1i8d | yes | 1 |
| 1i8t | yes | 1 |
| 1i9a | yes | 1 |
| 1idj | yes | 1 |
| 1idt | yes | 1 |
| 1iec | yes | 1 |
| 1ig8 | yes | 1 |
| 1im5 | yes | 1 |
| 1ima | yes | 1 |
| 1inp | yes | 1 |
| 1iph | yes | 3 |
| 1it4 | yes | 1 |
| 1itq | yes | 1 |
| 1itx | yes | 1 |
| 1iu4 | no |  |
| 1ivh | yes | 1 |
| 1iyd | yes | 1 |
| 1j00 | yes | 1 |
| 1j09 | yes | 4 |
| 1j2u | yes | 3 |
| 1j49 | yes | 1 |
| 1j53 | yes | 1 |
| 1j70 | no |  |
| 1j79 | yes | 1 |
| 1j7g | no |  |
| 1jch | no |  |
| 1jdw | no |  |
| 1jen | yes | 1 |
| 1jfl | yes | 1 |
| 1jh6 | yes | 1 |
| 1jhf | no |  |
| 1jkm | yes | 1 |
| 1jms | yes | 1 |
| 1joa | yes | 1 |
| 1jof | yes | 2 |
| 1js4 | yes | 2 |
| 1jxa | yes | 3 |
| 1jxh | yes | 1 |
| 1k0w | yes | 1 |
| 1k30 | yes | 1 |
| 1k32 | yes | 1 |
| 1k4l | yes | 1 |
| 1k4t | yes | 1 |
| 1k82 | yes | 1 |
| 1kae | yes | 1 |
| 1kas | yes | 1 |
| 1kaz | yes | 1 |
| 1kcz | yes | 1 |
| 1kdg | yes | 1 |
| 1kez | yes | 1 |
| 1kim | yes | 1 |
| 1kl7 | yes | 1 |
| 1knp | yes | 1 |
| 1kny | yes | 1 |
| 1kp2 | no |  |
| 1kqc | yes | 2 |
| 1kqf | no |  |
| 1ksj | no |  |
| 1kws | yes | 1 |
| 1kyq | no |  |
| 1kyw | yes | 1 |
| 1kzh | yes | 2 |
| 1kzl | no |  |
| 1l0o | yes | 1 |
| 1l1d | yes | 1 |
| 1l1l | yes | 1 |
| 1l1r | yes | 1 |
| 1l6p | no |  |
| 1l7d | yes | 1 |
| 1l7n | no |  |
| 1l7q | no |  |
| 1l8t | yes | 1 |
| 1l9x | yes | 1 |
| 1lam | no |  |
| 1lbu | yes | 1 |
| 1lcb | yes | 1 |
| 1lci | yes | 1 |
| 1ldm | no |  |
| 1lij | yes | 1 |
| 1lio | yes | 1 |
| 1lj1 | no |  |
| 1ljl | no |  |
| 1lml | no |  |
| 1lnh | no |  |
| 1ltq | no |  |
| 1luc | no |  |
| 1lvh | yes | 1 |
| 1lws | no |  |
| 1lxa | no |  |
| 1lya | yes | 1 |
| 1lz1 | no |  |
| 1m21 | yes | 1 |
| 1m53 | yes | 2 |
| 1m54 | yes | 4 |
| 1m6k | yes | 2 |
| 1m9c | yes | 1 |
| 1mas | yes | 1 |
| 1mfp | yes | 1 |
| 1mhl | yes | 1 |
| 1mht | yes | 1 |
| 1mj9 | no |  |
| 1mka | no |  |
| 1mla | yes | 1 |
| 1mlv | no |  |
| 1mok | no |  |
| 1moq | yes | 1 |
| 1mpx | yes | 1 |
| 1mpy | yes | 2 |
| 1mqw | yes | 1 |
| 1mro | no |  |
| 1mrq | yes | 1 |
| 1mt5 | yes | 1 |
| 1muc | yes | 1 |
| 1mud | yes | 1 |
| 1mug | yes | 1 |
| 1mvn | yes | 1 |
| 1myr | yes | 1 |
| 1n20 | no |  |
| 1n29 | yes | 1 |
| 1n2c | yes | 1 |
| 1n2t | yes | 3 |
| 1naa | yes | 1 |
| 1nba | yes | 2 |
| 1nbf | yes | 2 |
| 1ndh | yes | 1 |
| 1ndi | yes | 1 |
| 1ndo | yes | 4 |
| 1nf9 | yes | 1 |
| 1nhx | yes | 1 |
| 1ni4 | no |  |
| 1nid | yes | 1 |
| 1nir | yes | 1 |
| 1nkk | yes | 1 |
| 1nln | no |  |
| 1nlu | yes | 2 |
| 1nml | yes | 1 |
| 1nsf | yes | 1 |
| 1nsj | no |  |
| 1nsp | yes | 1 |
| 1nu3 | yes | 2 |
| 1nvm | no |  |
| 1nvt | yes | 1 |
| 1nww | yes | 1 |
| 1nzy | no |  |
| 1o04 | yes | 1 |
| 1o8a | yes | 1 |
| 1o9i | yes | 1 |
| 1oac | yes | 7 |
| 1oas | yes | 1 |
| 1oba | no |  |
| 1oe8 | yes | 1 |
| 1ofd | yes | 3 |
| 1ofg | yes | 1 |
| 1og1 | yes | 1 |
| 1oh9 | yes | 1 |
| 1oj4 | yes | 1 |
| 1ok4 | yes | 1 |
| 1okg | no |  |
| 1onr | yes | 1 |
| 1opm | yes | 1 |
| 1or8 | yes | 1 |
| 1ord | yes | 2 |
| 1oro | yes | 2 |
| 1os7 | yes | 2 |
| 1otg | yes | 1 |
| 1oxa | yes | 1 |
| 1oya | yes | 1 |
| 1oyg | yes | 1 |
| 1ozh | no |  |
| 1p1x | yes | 1 |
| 1p3d | yes | 1 |
| 1p4n | yes | 1 |
| 1p4r | no |  |
| 1pa9 | no |  |
| 1pad | no |  |
| 1pbg | yes | 1 |
| 1peg | yes | 4 |
| 1pfk | no |  |
| 1pfq | no |  |
| 1pix | yes | 6 |
| 1pja | yes | 1 |
| 1pjb | yes | 1 |
| 1pjh | yes | 3 |
| 1pjq | no |  |
| 1pkn | no |  |
| 1pma | yes | 2 |
| 1pnt | no |  |
| 1pow | no |  |
| 1pp4 | yes | 1 |
| 1ps1 | no |  |
| 1ps9 | yes | 2 |
| 1psd | yes | 1 |
| 1ptd | yes | 1 |
| 1pud | yes | 1 |
| 1pvd | no |  |
| 1pwh | yes | 1 |
| 1pwv | no |  |
| 1pxv | yes | 2 |
| 1pya | yes | 1 |
| 1pyl | no |  |
| 1pym | no |  |
| 1pz3 | yes | 3 |
| 1q18 | yes | 1 |
| 1q3n | yes | 1 |
| 1q3q | yes | 3 |
| 1q6l | yes | 1 |
| 1q6x | yes | 1 |
| 1q91 | yes | 1 |
| 1qam | yes | 1 |
| 1qaz | yes | 1 |
| 1qcn | no |  |
| 1qd1 | yes | 1 |
| 1qe3 | no |  |
| 1qf6 | no |  |
| 1qfe | yes | 1 |
| 1qfl | yes | 1 |
| 1qfm | no |  |
| 1qgn | yes | 1 |
| 1qgx | yes | 1 |
| 1qh5 | yes | 2 |
| 1qh9 | yes | 2 |
| 1qhf | yes | 1 |
| 1qhg | yes | 1 |
| 1qho | yes | 2 |
| 1qi9 | yes | 6 |
| 1qib | yes | 1 |
| 1qj4 | yes | 1 |
| 1qje | yes | 1 |
| 1qk2 | yes | 1 |
| 1qlh | yes | 1 |
| 1qmh | yes | 1 |
| 1qol | yes | 2 |
| 1qpr | yes | 1 |
| 1qq5 | yes | 1 |
| 1qrg | no |  |
| 1qrr | yes | 1 |
| 1qrz | yes | 1 |
| 1qsg | yes | 1 |
| 1qtn | yes | 1 |
| 1qum | yes | 1 |
| 1qv0 | yes | 1 |
| 1qx3 | no |  |
| 1qz9 | yes | 2 |
| 1r16 | yes | 1 |
| 1r1j | no |  |
| 1r30 | yes | 1 |
| 1r44 | yes | 1 |
| 1r4f | yes | 1 |
| 1r4z | no |  |
| 1r76 | yes | 2 |
| 1ra0 | yes | 3 |
| 1ra2 | yes | 1 |
| 1rba | no |  |
| 1rbl | yes | 1 |
| 1rbn | no |  |
| 1rdd | no |  |
| 1req | yes | 1 |
| 1rgq | yes | 1 |
| 1rhc | yes | 1 |
| 1rhs | yes | 1 |
| 1rk2 | yes | 1 |
| 1ro7 | yes | 1 |
| 1roz | yes | 1 |
| 1rpt | yes | 1 |
| 1rpx | yes | 1 |
| 1rql | yes | 1 |
| 1ru4 | yes | 1 |
| 1rvv | no |  |
| 1s20 | yes | 1 |
| 1s2k | yes | 1 |
| 1s3i | yes | 1 |
| 1s95 | yes | 1 |
| 1s9c | yes | 1 |
| 1ses | no |  |
| 1sll | yes | 3 |
| 1slm | yes | 2 |
| 1sme | yes | 1 |
| 1sml | yes | 1 |
| 1smn | yes | 1 |
| 1snn | yes | 1 |
| 1sox | yes | 4 |
| 1ssx | no |  |
| 1std | yes | 1 |
| 1t0u | yes | 1 |
| 1t4c | yes | 1 |
| 1t7d | yes | 1 |
| 1tah | no |  |
| 1tde | yes | 1 |
| 1tdj | no |  |
| 1teh | yes | 1 |
| 1thg | yes | 1 |
| 1tht | no |  |
| 1tml | yes | 1 |
| 1tmo | yes | 1 |
| 1tox | no |  |
| 1trk | no |  |
| 1tyf | yes | 1 |
| 1tys | yes | 1 |
| 1tz3 | yes | 1 |
| 1u3f | yes | 1 |
| 1u5u | yes | 1 |
| 1u7u | yes | 1 |
| 1u8v | yes | 1 |
| 1uae | yes | 1 |
| 1uaq | no |  |
| 1uas | yes | 1 |
| 1uch | no |  |
| 1uf7 | yes | 1 |
| 1ujn | yes | 1 |
| 1uk7 | yes | 1 |
| 1ula | no |  |
| 1un1 | yes | 1 |
| 1uok | yes | 1 |
| 1uqr | yes | 1 |
| 1uro | yes | 1 |
| 1ush | yes | 1 |
| 1uw8 | no |  |
| 1v04 | yes | 1 |
| 1v0e | no |  |
| 1v0y | no |  |
| 1v25 | no |  |
| 1vao | yes | 1 |
| 1vas | no |  |
| 1vid | yes | 1 |
| 1vie | no |  |
| 1vnc | no |  |
| 1vom | yes | 5 |
| 1vq1 | yes | 1 |
| 1vr7 | yes | 1 |
| 1vzx | yes | 1 |
| 1vzz | yes | 1 |
| 1w0h | yes | 1 |
| 1w1o | yes | 1 |
| 1w27 | no |  |
| 1w2n | yes | 2 |
| 1wd8 | no |  |
| 1wgi | yes | 1 |
| 1wnw | yes | 1 |
| 1x7d | yes | 1 |
| 1x9h | yes | 1 |
| 1x9y | yes | 2 |
| 1xa8 | yes | 2 |
| 1xgm | yes | 1 |
| 1xik | no |  |
| 1xny | yes | 1 |
| 1xqd | yes | 1 |
| 1xqw | yes | 2 |
| 1xs1 | yes | 1 |
| 1xtc | yes | 1 |
| 1xva | no |  |
| 1xvt | yes | 3 |
| 1xyz | yes | 1 |
| 1y9m | yes | 4 |
| 1ybq | no |  |
| 1ybv | yes | 1 |
| 1ycf | yes | 2 |
| 1ygh | yes | 1 |
| 1ylu | yes | 1 |
| 1yon | yes | 1 |
| 1ysc | yes | 1 |
| 1ytw | no |  |
| 1z9h | yes | 1 |
| 1ze1 | yes | 1 |
| 1zio | yes | 1 |
| 1zoi | yes | 1 |
| 1zrz | yes | 1 |
| 1zym | no |  |
| 206l | yes | 1 |
| 2a0n | yes | 1 |
| 2a86 | yes | 1 |
| 2aat | yes | 1 |
| 2abk | yes | 1 |
| 2ace | yes | 1 |
| 2acu | yes | 1 |
| 2acy | no |  |
| 2adm | yes | 1 |
| 2ag0 | no |  |
| 2ahj | yes | 1 |
| 2alr | no |  |
| 2amg | yes | 2 |
| 2apr | yes | 1 |
| 2ayh | yes | 1 |
| 2bhg | yes | 3 |
| 2bif | no |  |
| 2bkr | yes | 1 |
| 2bmi | yes | 1 |
| 2bsx | yes | 1 |
| 2bx4 | no |  |
| 2c7v | yes | 1 |
| 2cnd | yes | 1 |
| 2cpo | yes | 1 |
| 2cpu | yes | 1 |
| 2dbt | yes | 1 |
| 2dhn | no |  |
| 2dln | yes | 1 |
| 2dor | yes | 1 |
| 2dw7 | yes | 1 |
| 2ebn | no |  |
| 2eng | yes | 1 |
| 2eql | yes | 1 |
| 2esd | yes | 1 |
| 2f9r | yes | 1 |
| 2fmn | yes | 1 |
| 2fok | yes | 5 |
| 2fqq | no |  |
| 2gsa | yes | 1 |
| 2hdh | yes | 1 |
| 2hgs | yes | 1 |
| 2his | yes | 1 |
| 2hsa | yes | 1 |
| 2isd | no |  |
| 2jcw | no |  |
| 2jxr | yes | 1 |
| 2lip | yes | 1 |
| 2lpr | no |  |
| 2nac | yes | 2 |
| 2nlr | yes | 1 |
| 2nmt | no |  |
| 2npx | yes | 1 |
| 2oat | yes | 1 |
| 2pda | no |  |
| 2pec | yes | 1 |
| 2pfl | yes | 2 |
| 2pgd | yes | 2 |
| 2phk | yes | 1 |
| 2pia | yes | 1 |
| 2plc | yes | 1 |
| 2pth | yes | 1 |
| 2rnf | no |  |
| 2sqc | yes | 1 |
| 2tdt | yes | 1 |
| 2thi | yes | 1 |
| 2tmd | yes | 3 |
| 2toh | yes | 1 |
| 2tpl | yes | 1 |
| 2tps | yes | 1 |
| 2ts1 | yes | 1 |
| 2xis | yes | 1 |
| 2ypn | yes | 1 |
| 3cla | no |  |
| 3csm | yes | 1 |
| 3eca | no |  |
| 3mdd | no |  |
| 3nos | yes | 1 |
| 3pva | no |  |
| 3r1r | yes | 1 |
| 4kbp | no |  |
| 4mdh | no |  |
| 5cox | no |  |
| 5cpa | yes | 1 |
| 5eat | no |  |
| 5enl | yes | 1 |
| 5fit | yes | 1 |
| 5rsa | no |  |
| 7atj | yes | 1 |
| 7nn9 | yes | 1 |
| 7odc | yes | 2 |
| 8pch | no |  |
| 9pap | no |  |
